# Ultraviolet Superradiance from Mega-Networks of Tryptophan in Biological Architectures

**DOI:** 10.1021/acs.jpcb.3c07936

**Published:** 2024-04-19

**Authors:** N. S. Babcock, G. Montes-Cabrera, K. E. Oberhofer, M. Chergui, G. L. Celardo, P. Kurian

**Affiliations:** † Quantum Biology Laboratory, Howard University, Washington, D.C. 20060, United States; ‡ Institute of Physics, Benemérita Universidad Autónoma de Puebla, Puebla 72570, Mexico; § Lausanne Centre for Ultrafast Science, École Polytechnique Fédérale de Lausanne, Lausanne CH-1015, Switzerland; ∥ Department of Physics and Astronomy, Università degli Studi di Firenze, Florence 50019, Italy

## Abstract

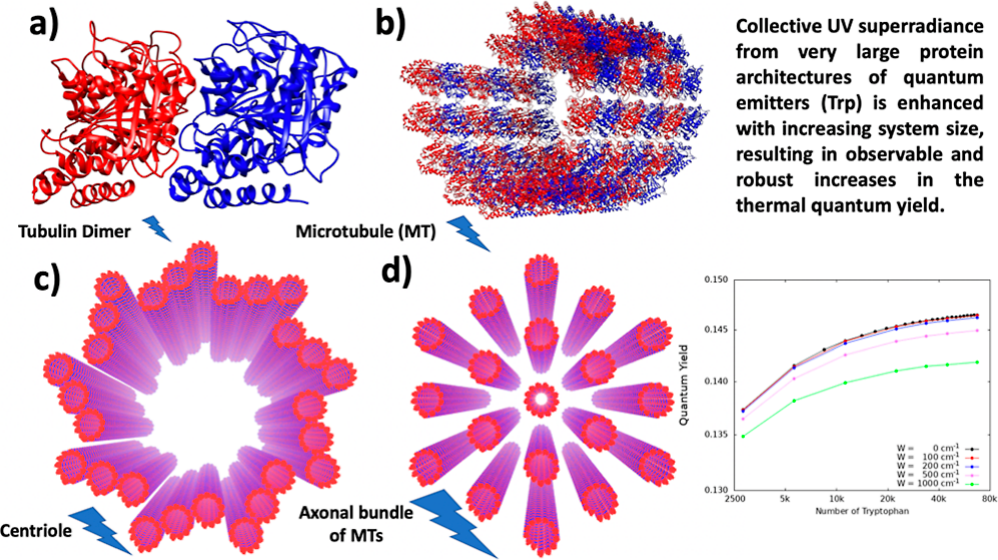

Networks of tryptophan (Trp)—an aromatic amino acid with strong fluorescence response—are ubiquitous in biological systems, forming diverse architectures in transmembrane proteins, cytoskeletal filaments, subneuronal elements, photoreceptor complexes, virion capsids, and other cellular structures. We analyze the cooperative effects induced by ultraviolet (UV) excitation of several biologically relevant Trp mega-networks, thus giving insights into novel mechanisms for cellular signaling and control. Our theoretical analysis in the single-excitation manifold predicts the formation of strongly superradiant states due to collective interactions among organized arrangements of up to >10^5^ Trp UV-excited transition dipoles in microtubule architectures, which leads to an enhancement of the fluorescence quantum yield (QY) that is confirmed by our experiments. We demonstrate the observed consequences of this superradiant behavior in the fluorescence QY for hierarchically organized tubulin structures, which increases in different geometric regimes at thermal equilibrium before saturation, highlighting the effect’s persistence in the presence of disorder. Our work thus showcases the many orders of magnitude across which the brightest (hundreds of femtoseconds) and darkest (tens of seconds) states can coexist in these Trp lattices.

## Introduction

Tryptophan (Trp) is the only amino acid with an indole moiety, making it a suitable precursor for a number of metabolites involved in biological signaling, most notably kynurenine and the neurotransmitter serotonin,^1^ which share Trp’s highly aromatic character. It is an ideal fluorescent reporter of biomolecular dynamics, given its natural occurrence in proteins, its strong ultraviolet absorption, and its significant absorption emission Stokes shift, which is highly sensitive to the protein, solvent, and electrostatic environments. As a matter of fact, in recent years, Trp has been used as a reporter of the Stark effect in photoactivated proteins,^2,3^ to monitor protein folding kinetics,^4^ as the operative chromophore in resonance energy transfer networks of UV-specific photoreceptor complexes,^5,6^ as a reporter of charge transfer states in proteins^7,8^ and of solvation dynamics at lipid–water and protein–water interfaces,^9,10^ to track local electrostatic changes in diverse classes of proteins,^11^ and as a probe for conformational ensembles of proteins in solution,^12^ among other applications.

Trp residues are often found in transmembrane proteins situated at the lipid–water interface. Multitryptophan proteins have been widely studied, including myoglobin, hemoglobin, cytochrome-*c* oxidase, and cytochrome *P*4̅50,^13^ as well as in the photoreceptors cryptochrome,^14^ bacteriorhodopsin,^2,3^ and UVR8.^5,6^ Large, organized Trp networks occur in these transmembrane proteins, receptors, and other macromolecular aggregates, lending essential structural and functional integrity to living systems.

**Figure 1:**
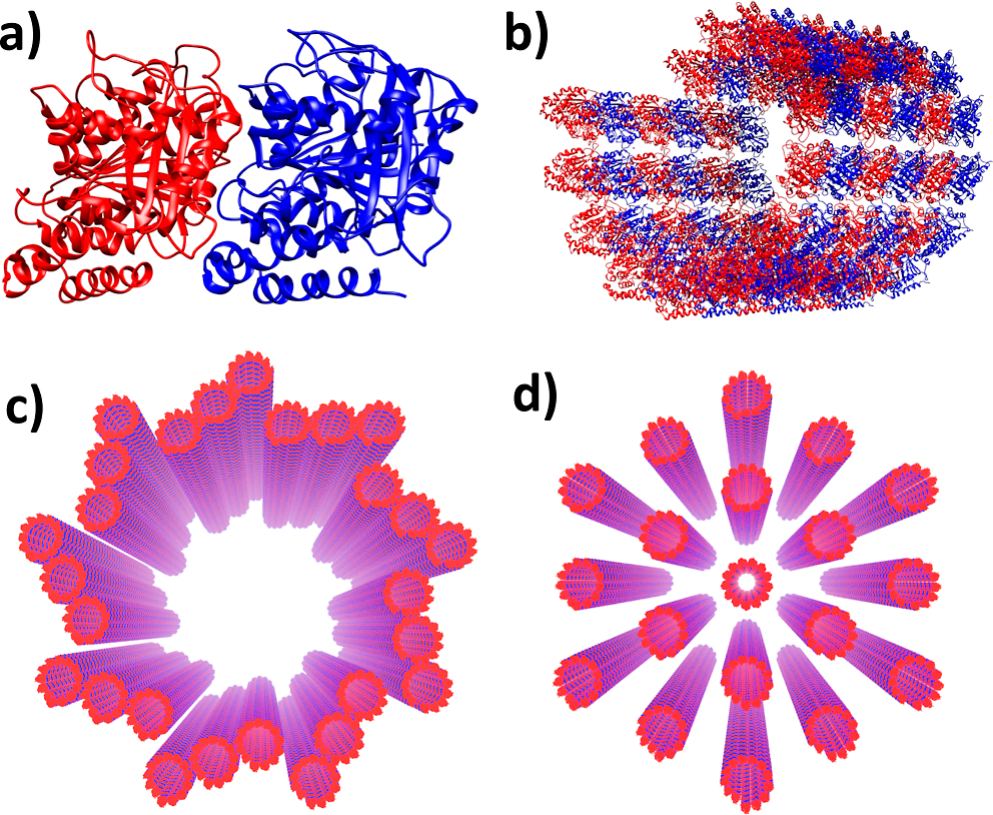
Hierarchical mega-networks of Trp form in protein architectures of functional biological significance. Panels depict a hierarchy of tubulin dimer structures composed of α and β tubulin (shown in blue and red, respectively), where panel (a) shows an individual TuD, (b) shows a MT segment of three dimer-defined spirals, (c) shows a centriole geometry formed from nine triplets of MTs, and (d) shows a hexagonal bundle of 19 MTs from a typical mammalian axon. Panels (a,b) were generated with *Chimera*. Panels (c,d) were produced using Visual Molecular Dynamics on the Argonne Leadership Computing Facility mainframe.

In particular, microtubules (MTs) are macromolecular aggregates of tubulin dimers (TuD) and represent mesoscale networks of Trp residues. MTs are spiral-cylindrical protein structures that self-assemble to enable cellular reorganization and remodeling for mitosis, differentiation, transport, habitat exploration, and apoptosis,^15^ and they have been found to reorganize structurally under UV irradiation.^16,17^ In addition, other evolutionarily conserved structures consist of MT architectures, including the centriole, a vortex arrangement generally made of nine “slats” of MT triplets (see Figure 1), which has been the subject of several studies^18−21^ examining the cellular orientations to a light stimulus.

These findings suggest the potential for photophysical and photochemical control of MT dynamics, which have been correlated with the regulation and partitioning of reactive oxygen species (ROS) in living cells.^22^ Endogenous, optical ultraweak photon emissions (UPEs) from living organisms are well-documented^23,24^ in the context of ROS-mediated oxidative stress. Stress-induced ultraviolet UPEs are more prominent during the exponential growth phase of the cellular cycle,^25,26^ implicating them in potential biophotonic signaling along aromatic networks during oxidative metabolism.^27^ However, the link among cellular metabolic activity, UPEs, and Trp network optical dynamics remains far from clear, leaving a critical gap in our knowledge.

Here, we explore the role of photoexcitation in mesoscale Trp networks present in several biological architectures. We show that mega-networks of Trp can exhibit a collective optical response in the UV region. By analyzing several architectures containing more than 10^5^ Trp chromophores—ranging from centrioles to MT bundles found in neuronal axons—we predict that strongly superradiant (paired with subradiant) states are often present in their spectra. Combining numerical results and scaling analysis, we determine the strength of the collective response in biological structures of realistic sizes. The effects of physiological disorder are considered by including fluctuations in the Trp excitation energies, demonstrating that the effects of superradiance can survive even at thermal equilibrium. Our predictions are confirmed by our experimental observations of larger quantum yields (QYs) with an increasing Trp network size.

## Materials and Methods

### Protein Structural Models

We created computer models of these realistic biological geometries using the atomic coordinates of proteins downloaded from the Protein Data Bank (PDB). We extracted the Trp coordinates (positions and orientations) from each PDB file to create tables of transition dipole moment coordinates as in ref (28), choosing the well-known ^1^L_a_ peak excitation at 280 nm as our transition dipole moment of interest.^2^

We used the Trp transition dipole coordinates obtained for each structure to define matrix elements of the radiative Hamiltonian given in eq S2 (see the Supporting Information and Figure S4 for further details). The complex eigenvalues of eq S3 in the Supporting Information contain information on the energies {*E*_*j*_} and radiative decay rates {Γ_*j*_} in the single-excitation limit.^28^ We simulated these resonances by diagonalizing the matrix in eq S3 for each Trp arrangement (see the Supporting Information). These spectra allowed us to predict enhancements due to collective quantum optical interactions in the Trp networks found in a variety of prototypical cellular structures, organelles, and appendages.

Tubulin (Figure 1a) was modeled using the PDB entry 1JFF, and tubulin dimers (TuD) were assembled into a virtual MT (Figure 1b) according to the protocol given in Appendix A of ref (28), aligning the (would-be) outer MT surface with the *y* axis (by rotating it counterclockwise 55.38° in the *yz* plane transverse to the MT longitudinal *x* axis) and optimizing the tubulin orientation (i.e., rotating each dimer clockwise 11.7° around the β-tubulin Trp346 C_δ2_ atom in the *yz* plane and then translating the dimer 0.3 nm in the *z* direction) before translating each dimer 11.2 nm in the *y* direction and successively rotating it clockwise by multiples of 27.69° in the *yz* plane (around the *x* axis) while successively shifting each dimer by multiples of 0.9 nm in the *x* direction.

Larger MT architectures were built virtually from arrangements of individual MTs, which were constructed as described above. MT bundles (from model axons as shown in Figures 1d and 6) were prepared in hexagonal arrangements by placing adjacent MTs 50 nm apart center-to-center, reflecting the mean separation between adjacent tau-mediated MTs in experimentally observed neuronal axons.^29^ The model centriole as shown in Figures 1c and 5 was created from an initial triplet of MTs centered 100 nm from the origin along the *y* axis—with the MTs in this triplet centered at (*x*, *y*, *z*) coordinates of (0, 87, −22.5167), (0, 100, 0), and (0, 113, 22.5167) in nm—before each triplet was rotated clockwise in increments of 40° around the *x* axis in the *yz* plane. The idealized (1JFF) model axoneme was constructed as 10 pairs of MTs, with each MT pair spaced 26 nm apart center-to-center. One MT pair is centered at the origin, and the remaining nine pairs are spaced evenly (40° apart) and centered at a distance of 98 nm from the origin, as shown in Figure S3.

**Figure 2:**
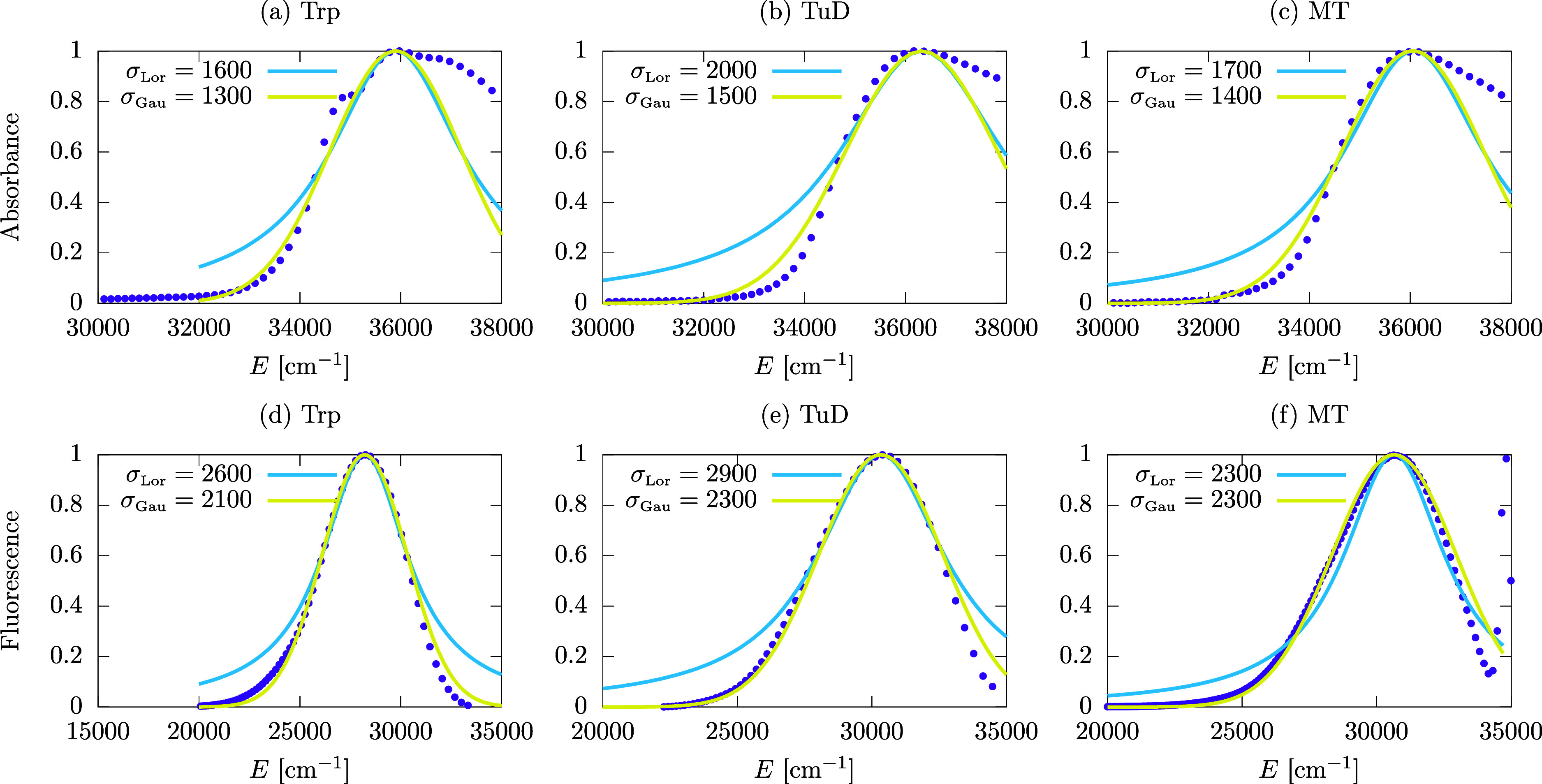
Absorption and fluorescence spectra for Trp, TuD, and MTs in aqueous solution. Comparison between experimental data (purple dots) and numerical estimates (solid curves). Two line shapes, Lorentzian (blue) and Gaussian (yellow), are considered for the numerical estimates. The homogeneous broadening is introduced by the parameter σ.

**Figure 3:**
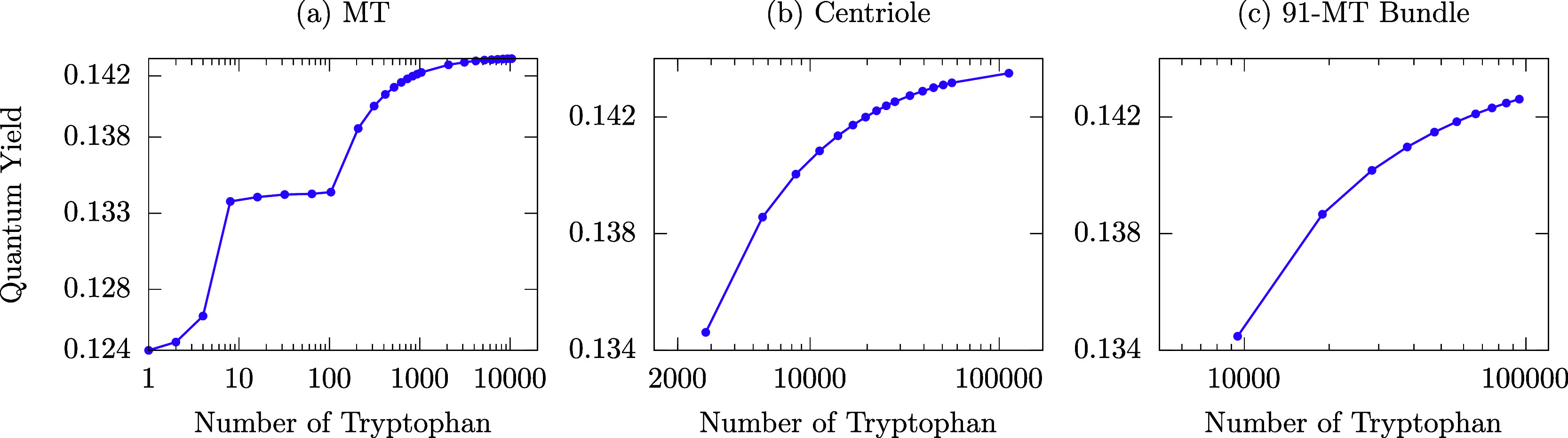
Predictions of fluorescence QYs from Trp networks in protein architectures assuming thermal equilibrium. The QYs are plotted as a function of the number of Trp chromophores, where panel (a) shows the QY for an MT (shown in Figure 1b) with a maximum length of ∼800 nm, panel (b) shows the QY for a centriole (shown in Figure 1c) formed by 27 MTs, each with a maximum length of ∼320 nm, and panel (c) shows the QY for a neuronal bundle formed by 91 MTs, each with a maximum length of ∼80 nm, arranged in a hexagonal honeycomb (similar to Figure 1d).

**Figure 4:**
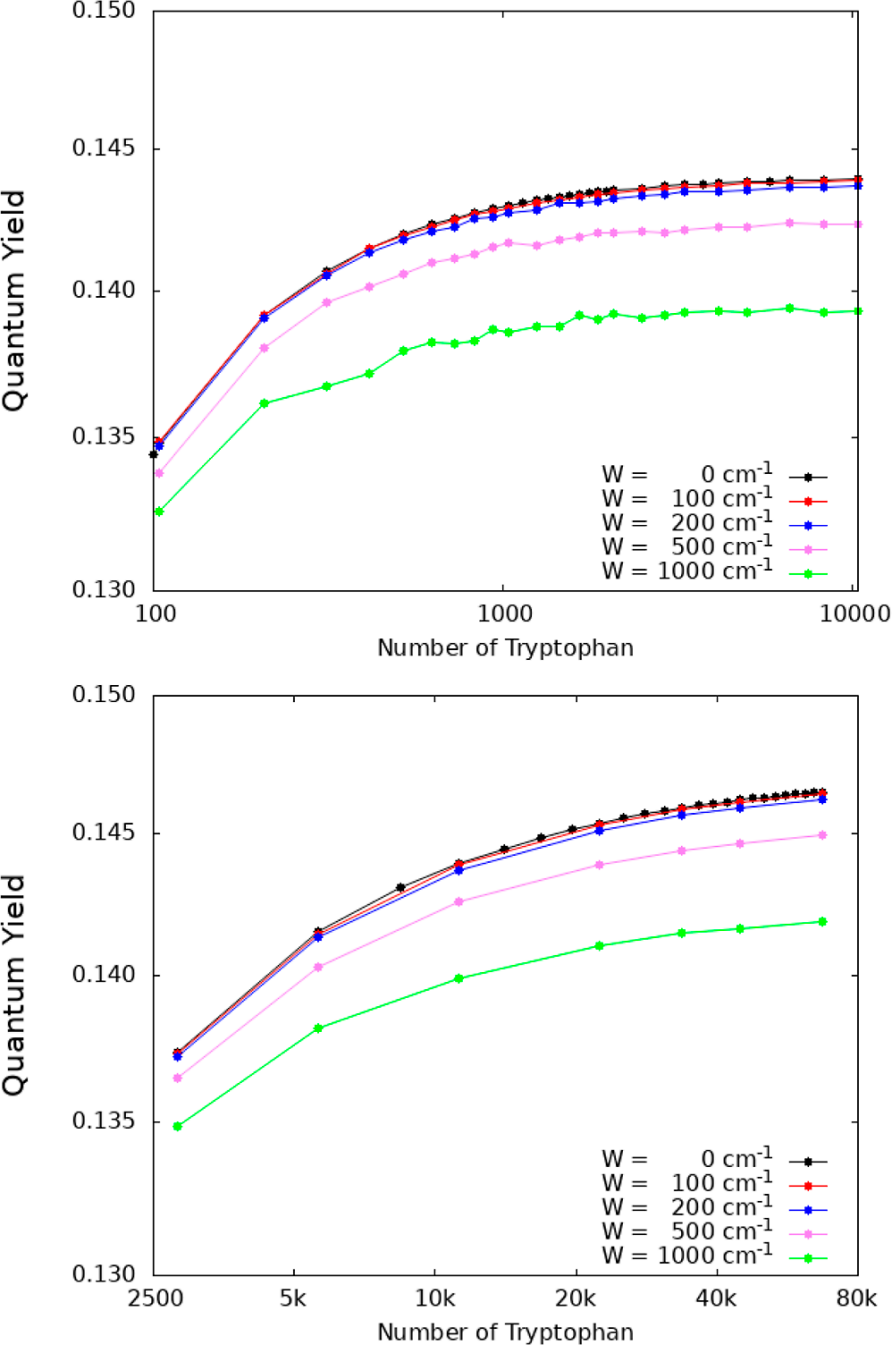
The fluorescence QY of large Trp lattices in protein architectures is robust to static disorder. The average of the thermal QY for 10 realizations of each static disorder strength *W* (see the legend), as a function of the MT (top panel) and centriole (bottom panel) length expressed by the number of Trps in each architecture, is shown. In the top (bottom) panel, a single MT (centriole) is considered with a maximum length of ∼800 (∼192) nm.

**Figure 5:**
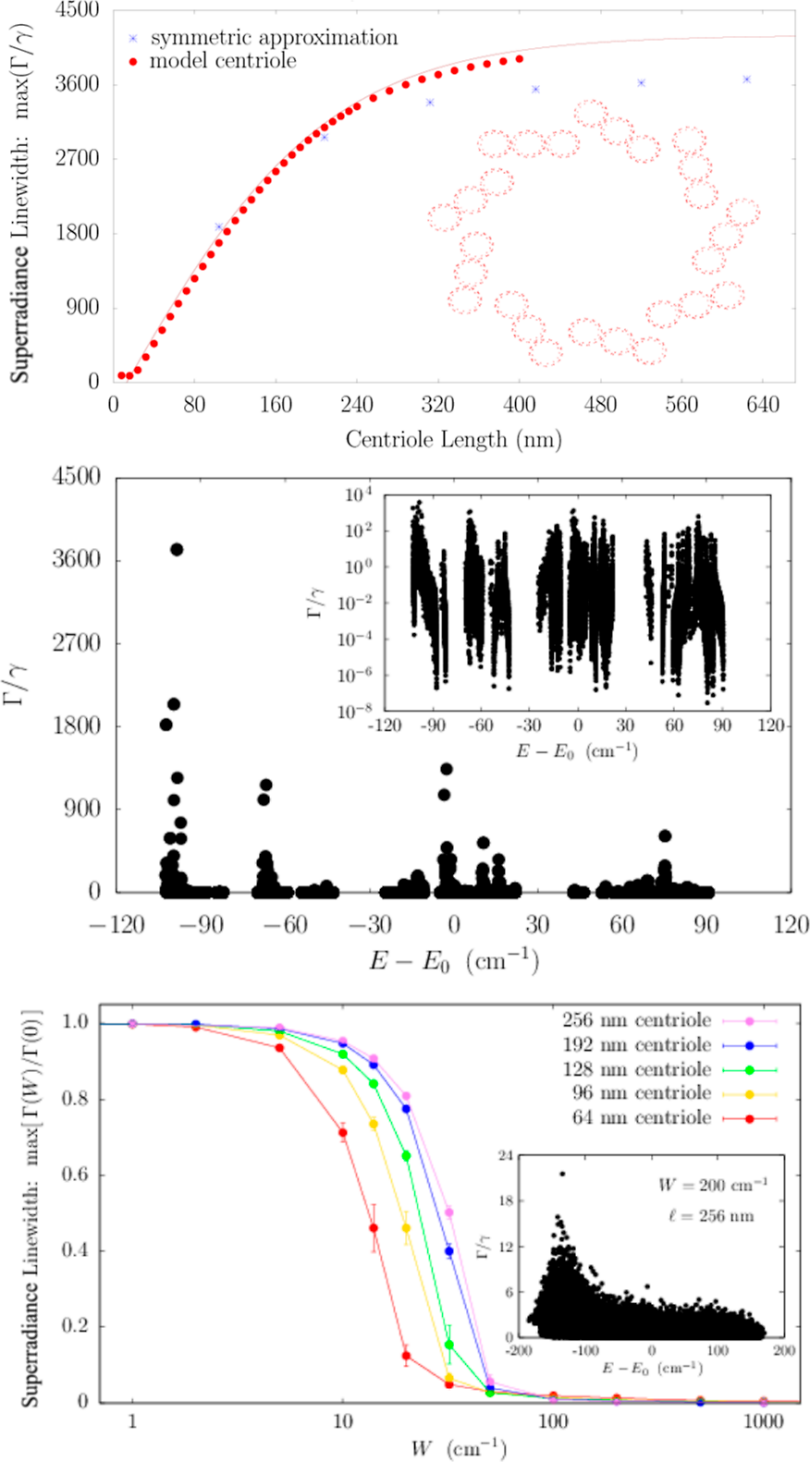
Prediction of superradiant states in the centriole and the robustness of superradiance to on-site disorder in centriolar Trp architectures. The top panel shows red superradiance data points max(Γ_*j*_/γ) calculated from numerical diagonalization of the radiative Hamiltonian in eq S3 for a model centriole Trp architecture, approximated by the curve , where  denotes the centriole length along its longitudinal axis (in nm),  denotes the longitudinal length of a single tubulin spiral, λ = 280 nm is the excitation wavelength, *n*_D_ = 8 is the number of Trp transition dipoles (^1^L_a_) per TuD, and *n*_S_ = 13 is the number of dimers per tubulin spiral. Blue stars represent the approximate predicted values for the brightest state given by eq 4. The top panel inset shows the centriole cross-section as point dipoles representing the Trp transition states. The center panel shows the spectrum of a 320 nm-long centriole containing 112 320 Trp dipoles, plotted on linear and semilog (inset) scales. The bottom panel shows superradiance data points with static disorder for model centrioles of lengths . The bottom panel inset shows the spectrum for a 256 nm long centriole containing 89 856 Trp dipoles at *W* = 200 cm^–1^ (i.e., commensurate with *k*_B_*T* at a temperature of *T* ≈ 288 K).

**Figure 6:**
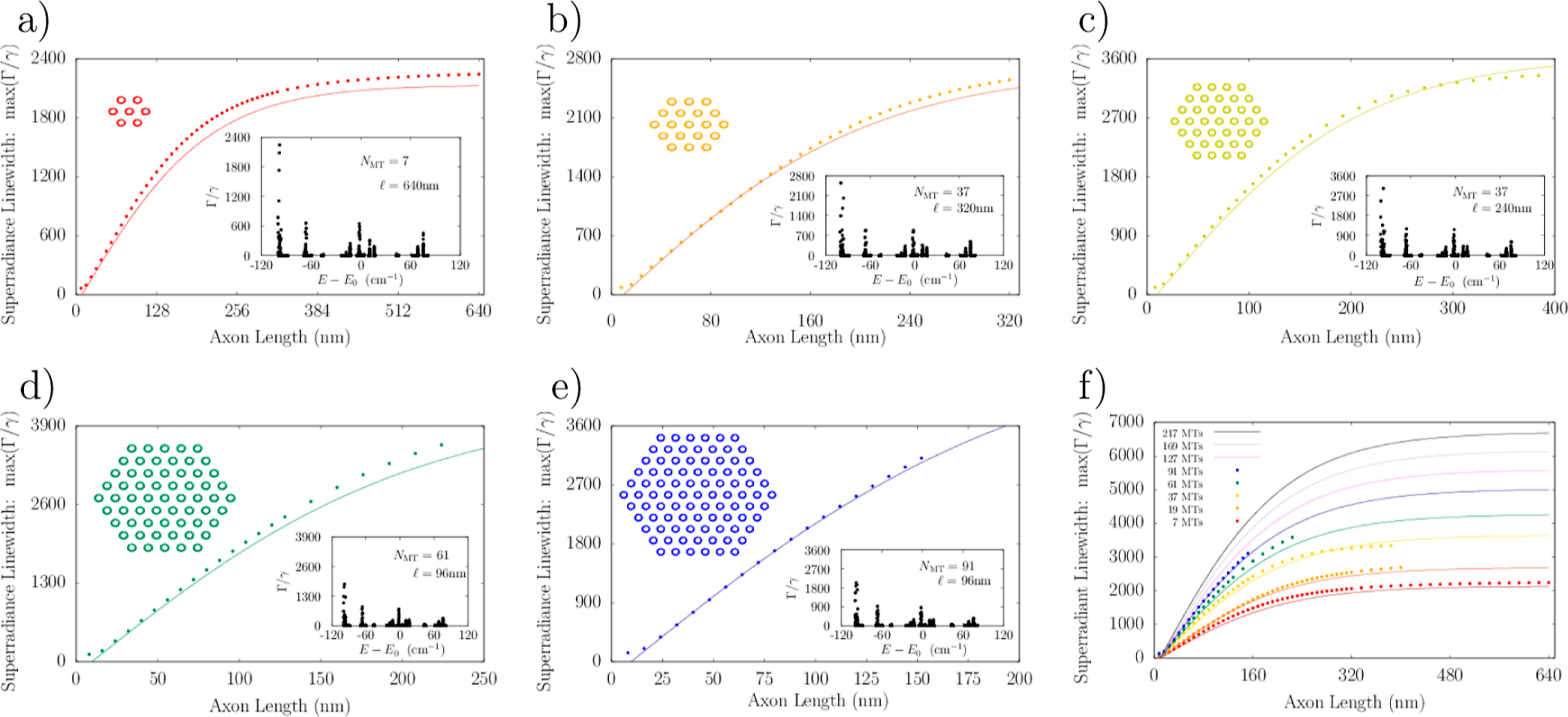
Neuronal MT bundles are predicted to exhibit exceptionally bright states that saturate in superradiance scaling as they approach micron lengths. Panels (a–e) show linear–linear scale plots of superradiance data max(Γ_*j*_/γ) for model axons (MT bundles in hexagonal honeycomb arrangement) of increasing length comprising *N*_MT_ ∈ {7, 19, 37, 61, 91} MTs, in that order. In each panel in (a–e), the main figure shows the exact max(Γ_*j*_/γ) in colored points, approximated by the curve , where  is the axon length (in nm) and *N*_MT_ gives the number of MTs it contains. *N*_0_ = 7 is the number of MTs in the smallest hexagonal bundle, _0_ = 8 nm is the length along the longitudinal axis of a single MT spiral, λ = 280 nm is the excitation wavelength, *n*_D_ = 8 is the number of Trp ^1^L_a_ transition dipoles per TuD shown in Figure 1a, *n*_S_ = 13 is the number of TuDs per MT spiral shown in Figure 1b, and *d* = 3 is the spatial dimension. The axon cross-section is shown as the inset in the upper left of each of the panels (a–e), and the complex spectrum (Γ/*γ* vs *E* – *E*_0_) for the longest numerically solved axon Trp architecture is shown in the lower right of each panel. Panel (f) summarizes the plots of the superradiance data max(Γ_*j*_/γ) and extrapolates to larger length scales using the analytical function above. The legend of panel (f) reflects the color scheme exhibited across panels (a–e). Red, orange, yellow, green, and blue solid curves reflect fits for *N*_MT_ = 7, 19, 37, 61, and 91, respectively, whereas violet, gray, and black curves predict data for axonal MT bundles with *N*_MT_ = 127, 169, and 217, respectively.

### Predictions of QY

The fluorescence QY is the ratio of the number of emitted photons (per time and volume unit) relative to the number of absorbed ones. Equivalently, the QY can be defined in terms of the radiative decay rate Γ and the nonradiative decay rate Γ_nr_: QY = Γ/(Γ + Γ_nr_), where Γ_nr_ represents all of the physical and chemical processes involved in the interaction between the chromophore network and the surrounding protein(s) or solvent. Typically, we can write Γ_nr_ = Γ_IC_ + Γ_ISC_ + Γ_react_, where Γ_IC_ is the internal conversion rate constant; Γ_ISC_ is the intersystem crossing rate constant; and Γ_react_ is the rate constant due to quenching or photochemical reactions.

The effective Hamiltonian  from eq S3 in the Supporting Information is the starting point for our QY predictions. To consider the effects of nonradiative processes in our model, we replace the diagonal part of  with a new decay rate γ′ = γ + γ_nr_. Here, γ_nr_ represents the decay rate of a single Trp due to nonradiative processes. Then, the new eigenvalues of  are given by  where Γ_*j*_^′^ = Γ_*j*_ + Γ_nr,*j*_. The QY is a dimensionless quantity that takes values between 0 and 1. When Γ ≫Γ_nr_, QY → 1, but if the excited state depopulation is dominated by quenching processes, external conversion, or intersystem crossing, Γ_nr_ ≫Γ and QY → 0.

For the particular case of the Trp chromophore, the radiative decay rate γ = 0.00273 cm^–1^ corresponds to a radiative lifetime of τ = 1.9 ns.^28^ At room temperature, its QY in water is estimated to be QY ≈ 0.13,^30^ although in different proteins, the Trp QY has been observed to vary from about 1/10 this value to nearly a factor of 3 times it.^7^ As a standard for comparison with Trp in tubulin and MTs, we used our experimentally measured value of 12.4% for the QY of Trp in BRB80 buffer, which was also used for the protein solutions. Using eq 3 with the replacements Γ → γ and Γ_nr_ → γ_nr_ allows us to calculate the Trp nonradiative decay rate in water as γ_nr_ ≈ 0.0193 cm^–1^.

### Thermal Average

Consider *P*(*t*) as the probability that an excitation is found in the chromophore network at time *t*, while 1 – *P*(*t*) would be the probability that the excitation has left the network. Let us denote as *P*_k_(*t*) the probability that the chromophore system is described by the eigenstate  at time *t*. Assuming thermal equilibrium, *P*_k_(*t*) = *P*(*t*) exp(−*E*_k_/*k*_B_*T*)/*Z*. Here, *k*_B_ = 0.695 cm^–1^ K^–1^ stands for the Boltzmann constant, *T* is the temperature, and *Z* = ∑_*j* = 1_^*N*^exp(−*E*_*j*_/*k*_B_*T*) is the partition function.

Due to the non-Hermitian nature of , the probability  at thermal equilibrium is not conserved . Instead, it satisfies the following master equation, , where the thermal average for the decay rate is given by ⟨Γ⟩_th_ = ∑_*j* = 1_^*N*^Γ_*j*_exp(−*E*_*j*_/*k*_B_*T*)/*Z*. Because γ_*j*_ for each Trp is assumed equal to all others and our model neglects the formation of additional nonradiative channels with the increasing Trp network size, we consider  and arrive at the following definition for the QY at thermal equilibrium
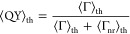
1

### Measurements of QY

Steady-state fluorescence spectroscopy was performed with a Shimadzu RF-5301PC spectrofluorophotometer. Conventional absorption spectra were obtained with a Shimadzu UV-3600 UV–vis spectrophotometer. For the measurements, we used UV-grade glass cuvettes with a path length of 1 cm.

#### Samples

Tubulin protein in the form of α–β tubulin heterodimers and preformed MTs (taxol-stabilized and lyophilized) extracted from porcine brain were purchased from Cytoskeleton, Inc. The MTs exhibit an average length of 2 μm. For tubulin and Trp (Sigma-Aldrich) solutions, we used a self-prepared BRB80 buffer (80 mM PIPES pH 6.9, 2 mM MgCl_2_, and 0.5 mM EGTA, pH 6.9). MTs were stabilized in solution by adding 20 μM taxol (Cytoskeleton, Inc.) to the BRB80 buffer. The proteins are delivered as powders in 1 mg vials (TuD) and 0.5 mg vials (MTs). However, the vials contain a bit more than 1 and 0.5 mg, respectively, according to the manufacturer. Moreover, around 5 mg of sucrose plus 1 mg of Ficoll is added to the vials. Hence, it was not possible for us to prepare solutions with exact concentrations. We used ∼0.33 mg/mL tubulin protein and MTs for steady-state spectroscopy. Tyrosine was measured in ultrapure water solution and cysteine was measured in 1 mM HCl. The absorption background by the solvents was subtracted.

#### Absorption and Emission Spectra

Steady-state absorption and emission spectra of MTs, tubulin protein (α–β tubulin heterodimers, TuD), and Trp in physiological BRB80 buffer are shown in Figures 2 and S2. They reveal a first absorption band with a maximum at around 280 nm for all three systems. The MT solution exhibits a strong scattering background (Figure S2d), which needed to be subtracted. Assuming Rayleigh-like scattering (∝ λ^–4^), we fitted the background from 307 to 800 nm. The extrapolated curve for wavelengths λ < 307 nm was subtracted from the raw spectrum. The corrected spectrum agrees qualitatively well with the TuD spectrum for wavelengths above ∼270 nm. The upper limit for the MT QY is determined from the corrected spectrum (by subtracting the fit, resulting in a lower sample absorbance *a*_s_ in eq 2), and the lower limit for the MT QY is determined from the raw data without correction.

The normalized fluorescence spectra upon excitation at 280 nm show that Trp fluorescence has its maximum at 355 nm, while for TuD and MTs, the absorption emission Stokes shifts are significantly smaller than those for Trp and almost identical for both (fluorescence maxima at ∼327 nm). We attribute it to a reduced chromophore–solvent interaction due to the protein environment. The full-width half-maximum (fwhm) intensity values of TuD and MTs are experimentally identical (∼5.5 × 10^3^ cm^–1^) and are broader than that for Trp (4.9 × 10^3^ cm^–1^).

Each TuD includes 8 Trp, 35 tyrosine (Tyr), and 20 or 21 cysteine amino acids that can form up to 10 cystine (Cys) residues linked in pairs by disulfide bonds, even though most of these are too far apart to form such a bridge. In order to roughly estimate the molar absorption coefficient of the protein at ∼280 nm, we simulated the absorption spectrum of TuD by adding the contributions of Trp (5.6 × 10^3^ M^–1^ cm^–1^, 49% contribution), Tyr (1.3 × 10^3^ M^–1^ cm^–1^, 50% contribution), and Cys residues (125 M^–1^ cm^–1^, 1% contribution), i.e., the number of the respective residues multiplied with their respective molar absorption coefficients at ∼280 nm.^31^ This yields a molar absorption coefficient of tubulin of 92 × 10^3^ M^–1^ cm^–1^. The reconstructed spectrum is in good agreement with the experimental one down to 270 nm (see Figure S2c). However, the experimental spectrum is slightly broadened, probably due to inhomogeneous contributions. The other amino acids from the protein’s backbone have negligible contributions (≪1%) to the molar absorption coefficient in this range. The deviations below 270 nm are presumably due to contributions of the more than 800 remaining amino acids of the protein backbone including, e.g., phenylalanine (43 residues) or alanine (80 residues).

Among these amino acids, Trp and Tyr exhibit the strongest fluorescence QYs (Trp 13% and Tyr 14% in H_2_O^32^). The fluorescence maximum of TuD is blue-shifted by 2.2 × 10^3^ cm^–1^ with respect to that of Trp and red-shifted by 2.9 × 10^3^ cm^–1^ with respect to that of Tyr (see Figure S2b). Hence, reconstruction of the TuD fluorescence spectrum by adding the spectra of the two components, even with optimized weights, is not possible.

#### Observation of Fluorescence QYs

Fluorescence QYs (QYs) were determined according to the standard reference formula^33^
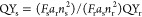
2

The subscripts *s* and *r* represent the examined sample and reference, respectively. *F* is the integrated fluorescence area and *n* is the refractive index of the respective solution. The absorption factors *a* are determined by *a* = 1–10^–*A*^, with *A* being the optical density at the absorption wavelength of the absorption band. The QY of Trp in H_2_O (∼13%) was used as in refs (30 and 33). We adjusted the concentrations of the samples to approximately equal optical densities in the absorption maxima of the various samples. In order to minimize statistical errors, the spectra of five freshly prepared solutions for each sample were averaged.

The QY of Trp in the BRB80 buffer (12.4 ± 1.1%) does not deviate significantly from its value in H_2_O. Tubulin exhibits a QY of 6.8 ± 0.4%, which is reduced with respect to that of Trp. This indicates the contribution of other chromophores to the absorption band, on one hand, and the dominant Trp fluorescence, on the other hand. Lacking knowledge of the exact contribution of scattering to the optical density of the MT solution at 280 nm, we can give only a range or average for the QY of MTs. By subtracting the extrapolated fit from the raw data, we estimate 12.0 ± 1.0% as an upper limit of the MT QY. Its lower limit is given by the MT absorption without any scattering corrections and yields 10.3 ± 0.8%.

In order to determine the QY generated by the Trp residues in TuD and in MTs, we weighted the absorption spectrum by its Trp contribution, which is 49% at 280 nm. Therefore, the QY for TuD grows to 10.6 ± 0.6%, and for MTs, we get 19.5 ± 2.8% for its upper limit and 15.7 ± 1.3% for its lower limit (Table 1 of the main text and Table S2). TuD still exhibits a reduced QY with respect to Trp, but the MT QY from Trp is clearly enhanced.

**Table 1:** Fluorescence QYs from Trp Networks in Protein Architectures^a^

sample	QY-Trp @ 280 nm (%)	QY-Trp @ 295 nm (%)
MT	17.6* ± 2.1	14.7* ± 1.6
TuD	10.6 ± 0.6	10.9 ± 1.3
Trp	12.4 ± 1.1	11.4 ± 1.1

a Summary of experimental measurements obtained from steady-state spectroscopy of Trp, TuDs, and MTs in BRB80 aqueous buffer solution (see Figures 2, Table S2, and Figure S2 for complete spectral data). The fluorescence QY is determined for excitation at 280 and 295 nm (see the Materials and Methods section for details about the procedure). Note the statistically significant increases in the QY from tubulin to MTs, in qualitative agreement with Figure 3a and consistent with what one would expect in the presence of superradiance. The * indicates an average of upper and lower limit values for MTs, which have been corrected for the scattering background.

In order to minimize the error due to overlapping absorption by other residues, we also evaluated the QY at 295 nm (instead of the absorption maximum at 280 nm) excitation, where the absorption of amino acids other than Trp is negligible. The values are also given in Table S2, and they confirm the trends obtained for the 280 nm excitation.

## Results: Simulations and QY Measurements

We modeled biologically realistic arrangements of Trp chromophores, beginning with hierarchical aggregates of the protein tubulin, as shown in Figure 1 and as described in the Materials and Methods section. We then modeled photoemissive decay channels using a well-known radiative non-Hermitian Hamiltonian for open quantum systems (see the Supporting Information and refs (34 and 35) for further details). We thus characterized the collective light–matter interaction of Trp mega-networks present in several biologically relevant architectures, solving the complex eigenvalues *E*_*j*_ – iΓ_*j*_/2 for each Trp network geometry and determining the radiative decay rates Γ_*j*_ of the network eigenmodes. Comparing the maximum Γ_*j*_ with the radiative decay rate γ of a single Trp chromophore, we determined the superradiance enhancement factor max(Γ_*j*_)/γ, thus characterizing each architecture’s spectrum by its brightest (i.e., most superradiant) state. Such a model has allowed us to investigate the possibility of whether quantum optical modes may be implicated in the photonic coordination of the cytoskeleton and other cellular structures characterized by mesoscale networks of Trp.

The radiative and nonradiative decay processes of the emissive state, in our case, the ^1^L_a_ state of Trp (see the Supporting Information for further details on distinctions from the ^1^L_b_ state), are quantitatively described by their decay rates Γ and Γ_nr_, respectively.^32^Figure 2 shows the absorption and emission spectra of Trp, TuDs, and MTs. The absorption emission Stokes shift is almost identical for TuD and MTs and is significantly smaller than that of Trp. This implies, for the protein architectures, an overlapping resonance regime between absorption and emission around 300 nm, where the absorptive and emissive transition dipoles are resonant and experimentally indistinguishable. Qualitatively, the measured absorption maxima are almost the same for Trp, TuD, and MTs. The shapes of the absorption bands on the low-energy sides and the emission maxima for TuD and MT also are essentially the same, and both of these emission peaks occur at energies higher than that of Trp. The measured emission spectra of TuD and MT both are broader than those of Trp. In the prior work, again considering only the excitonic (and not mechanical) degrees of freedom, we predicted that the absorption bands would sharpen and shift to lower energy in MTs.^a^Figures S3, S5, and S6 show similar calculated shifts and heightened sharpening in other larger assemblies of MTs in the absence of disorder. Indeed, MTs do have a slightly sharper measured emission spectrum and a slightly lower peak absorption peak than TuD, which agrees with our model. However, though the small magnitudes of the shift and sharpening relative to the inhomogeneous broadening of the spectra ostensibly weigh against the notion that these effects have any physiological significance, we show below and in the next section how such incremental changes at the level of the MT can have robust consequences on the QY. Certainly, this should be considered distinct from the situation in photosynthetic systems, where more strongly coupled interactions between the pigment chromophores cause much larger changes in the absorption and emission spectra and where the excitation wavelengths lie in the visible range.

The emissive process is mainly characterized by the observed fluorescence lifetime and QY. The QY is defined as the ratio of the number of photons emitted to the number of photons absorbed, or equivalently
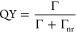
3

We predict the trends of steady-state QYs in the various Trp networks by calculating the thermal averages ⟨Γ⟩_th_ and  of the radiative and nonradiative decay rates, respectively, by means of the complex eigenvalues of the effective Hamiltonian in eq S3 (see the Supporting Information for further details).

Analyzing various biological architectures of Trp, we found the emergence of strong superradiant states close to the lowest excitonic state (see the Supporting Information). The superradiance enhancement increases with the system size until approximately a few times the excitation wavelength, and then, it tends toward saturation. The presence of a strong superradiant state close to the lowest-energy state (see Figures S3, S5, and S6) is expected to enhance the QY since the thermal occupation probability of such a superradiant state—and thus the thermally averaged radiative decay rate—will be enhanced.

Figure 3 shows the QY predictions for MTs (Figure 1b), centrioles (Figures 1c and 5), and 91-MT bundles (Figure 6e) of varying lengths. The QY is calculated and displayed in Figure 3 in the form of semilog plots as a function of the number of Trp chromophores in the network. Here, thermalization is assumed at *k*_B_*T* ≈ 207 cm^–1^, where Boltzmann’s constant is given by *k*_B_ = 0.695 cm^–1^ K^–1^ and the room-temperature bath is given by *T* = 298 K.

Figure 3a shows the case of an MT. Starting with an established experimental value for the QY of Trp,^30^ the MT QY behavior is divided into three regimes. The first one exhibits a rapid, but overall modest increase (<10%) corresponding to the formation of a single TuD, containing a total of eight Trp chromophores. The second regime shows near constancy (to 0.1%) corresponding to the formation of the first MT spiral layer. Each spiral layer contains 13 TuD and a total of 104 Trp chromophores (Figure 1b). This near-constant regime in the first MT spiral layer can be explained by the fact that the superradiant state is not close to the lowest-energy state for the first spiral, as shown in a previous work.^28^ The last regime for QY > 0.134 shows a familiar sigmoid-like increase and corresponds to the formation of the MT by adding one spiral layer after another, until 100 layers (∼800 nm) are reached, with a total of 10 400 Trp chromophores. QY saturation begins to set in when the MT has reached the length of a few λ, where 280 nm is the relevant scale set by the wavelength of incident light considered for excitation of the Trp chromophores. Such saturation in the QY is explained by the behavior of the superradiance enhancement factor, which also saturates at this length scale for a variety of structures containing Trp mega-networks (see Figures S3, S5, and S6).

Figure 3b shows the case of a centriole. The minimum length we consider is a single centriole layer of about 8 nm containing 2808 Trp chromophores. At 40 layers, we obtain a centriole approximately 320 nm in length and containing a total of 112 320 Trp chromophores. As long as the centriole volume contains between 3000 and 20 000 Trp chromophores, a rapid growth of QY is observed. For larger volumes, the growth of QY slows, but the saturation regime is still not fully realized. Figure 3c shows the case of a 91-MT bundle with 10 layers and a length of approximately 80 nm. The primary difference in this case is that each layer of the bundle contains 9464 Trp chromophores. Similar to the centriole case, the QY increases monotonically as chromophores are added to the network without realizing saturation even at 10^5^ Trps.

All three panels in Figure 3 are consistent in showing how thermalization significantly competes with enhancements to the QY from collective effects, without eliminating them, as the superradiance exhibited by these mega-networks in the absence of disorder varies from a few hundreds to several thousands of times the Trp spontaneous emission rate. In panel 3c, for example, by increasing the number of chromophores by 1 order of magnitude from 10^4^ to 10^5^, the increase in thermal QY is only ∼1%.

These results taken together suggest that equilibrium thermal effects are a primary cause of mitigating such cooperative quantum behaviors without entirely washing out the associated phenomena. Indeed, we also considered the effect of structural disorder on the thermal QY by adding time-independent fluctuations of the excitation energies of the Trp chromophores. These fluctuations are typically used to simulate inhomogeneous broadening of the absorption and emission spectra.^34^ Interestingly, we found that the QY is almost unaffected when a disorder strength equal to room-temperature energy (∼200 cm^–1^) is considered, and a QY enhancement is still observable even at 1000 cm^–1^ disorder (see Figure 4). Thus, the QY enhancements presented in Figure 3 are very robust to both thermal environments and structural disorder.

In order to verify the above theoretical predictions, we performed steady-state fluorescence QY measurements using the QY of Trp in water^30^ as a standard. The QYs were determined both for 280 nm excitation, subtracting contributions from other residues, and for 295 nm excitation, where only Trp absorbs (see the Supporting Information for further details). The QY measurements could only be performed on tubulin and MTs because of issues with scattered light and sample purity for the larger assemblies, as explained in the Supporting Information. Figure S1 shows the steady-state absorption and emission spectra of Trp, TuD, and MTs. A scattering background affects the absorption spectrum of MTs and was corrected for as explained in the Supporting Information. Table 1 shows consistent results for both excitation wavelengths, with the first a decrease of the QY from Trp to TuD and then a statistically significant increase by up to almost 70% for MTs. The decrease from Trp to TuD is small but non-negligible, suggesting that nonradiative processes in the protein are at play. However, this notwithstanding, the significant increases from TuD to MTs are in qualitative agreement with our predictions in Figure 3a, bearing in mind that our model does not account for additional nonradiative channels due to the formation of large Trp ensembles. It is noteworthy that the increased QY in MTs would imply a decreased nonradiative decay rate, but this is not the case with TuD—rather the contrary. It is therefore an unlikely scenario in the vastly more complex MT case, suggesting that collective radiative processes in these protein assemblies with mega-ensembles of Trp are the primary cause of the significant QY increases observed in MTs.

These promising results point toward superradiant emission, but fluorescence QYs need to be complemented by lifetime measurements in order to measure the radiative rate of fluorescence. This was not done in the present study as it requires measurements over a wide temporal range from femtoseconds to nanoseconds, which is not possible with a single experimental setup. In addition, even if such measurements were carried out, the fluorescence decays are nonexponential,^7,36^ and we cannot obtain a reliable radiative rate.

### Robustness of QY to Disorder

In this section, we analyze the robustness to static disorder of the QY enhancements presented above. Here, we consider how time-independent fluctuations of the Trp excitation energies, which are commonly attributed to interactions with distinct local environments, can affect the QY dependence on the Trp network size. To account for the influence of structural disorder, we considered time-independent fluctuations in the on-site energies of each Trp transition dipole using a random uniform distribution of energies within the range given by *E*_0_ ± *W*/2 for each emitter, introducing *W* as the disorder strength parameter. The QY was computed for each realization of disorder and then averaged over 10 realizations for each Trp network. Alternatively, one can average over multiple realizations of disorder *W* the thermally averaged radiative decay rate ⟨Γ⟩_th_ and then use eq 1 in the Materials and Methods section of the main text to compute the QY in the presence of structural disorder. We checked that both methods gave very similar results.

In Figure 4, we show the cases of a single MT (top panel) and a centriole (bottom panel). As one can see, the QY enhancement is extremely robust to static disorder. Indeed, even for a static disorder strength of the same magnitude as room-temperature energy (200 cm^–1^), the QY dependence on the MT and centriole lengths is basically unaffected.

The robustness of the QY to structural disorder is remarkable, given that such disorder strongly suppresses the superradiance enhancement factor. For instance, in the case of a centriole, the superradiance enhancement goes from ∼3600 in the absence of disorder to ∼20 for *W* = 200 cm^–1^ (see Figure 5). Nevertheless, the values of the QY for *W* = 0 cm^–1^ and *W* = 200 cm^–1^ are very close to each other. The origin of this robustness can be explained as follows: in the presence of static disorder, the superradiant dipole strength gets distributed among other excitonic states, but states close to the superradiant state in energy will still exhibit most of the dipole strength if the disorder is not overwhelming. If in the absence of disorder the superradiant state is close to the lowest excitonic state and in the presence of disorder its dipole strength gets distributed within *k*_B_*T* from it, then the QY is not affected drastically. This means that the QY is a very robust figure of merit for cooperativity. These results strengthen our prediction that an enhancement of the QY will persist even under ambient conditions in biological systems.

### Simulations of Centriole Superradiance

Dynamic organization of the MT network is coordinated in vertebrate cells from the centrosome,^37^ which is composed of two perpendicular centrioles^38^ in most eukaryotes. The *centriole* (Figure 1c) is one of the largest (protein-based) structures of the cell, exhibiting a cartwheel-like arrangement of MTs that can be as large as 250 nm in diameter and up to 500 nm in length in vertebrates.^38^ It is a pinwheel-shaped, barrel-like organelle that coordinates cellular orientation and division processes.

We modeled a prototype centriole of increasing length as an array of nine MT triplets, where each simulated MT was generated from the structure of the tubulin PDB entry 1JFF(39) following the past work.^28^ We numerically solved the spectrum of each centriolar Trp arrangement to predict the enhancement factors max(Γ_*j*_/γ) shown in the top panel of Figure 5. We found that the maximum superradiance enhancement increased with growing length until saturating at max(Γ_*j*_/γ) ≈ 4000, in the realistic length scale of vertebrate centrioles. This large superradiance enhancement is identified with a state in the lowest band of excitonic eigenstates of the Hamiltonian of eq S2, as displayed in the center panel of Figure 5 for a 320 nm centriole Trp architecture, where the variation in the energies spans about *E*_0_ ± 100 cm^–1^.

We developed an analytical approximation for the quantum state with the largest value of Γ (i.e., the most superradiant) state of each centriole, modeled as a weighted superposition of the most superradiant states of a set of 104 nm (13-spiral) MT segments, using the expression
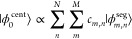
4where |ϕ_0_^cent^⟩ is the bioorthogonally normalized^40^ estimate of the most superradiant centriole state, M = 27 is the number of MTs comprising a centriole, and *N* is the number of 104 nm MT segments comprising the length of the centriole (e.g., *N* = 2 for a 208 nm long centriole). The indices *m* and *n* thus designate the locations of each segment state |ϕ_*m*,*n*_^seg^⟩, in effect defining the 27*N* MT segments making up the centriole. The real-valued coefficients are defined as
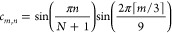
5where ⌈*x*⌉ denotes the ceiling function (i.e., the nearest integer larger than *x*). Although inexact, this approach allowed us to validate our fit with an estimate of the complex expectation value of the approximate superradiant state at a computational cost of  numerical operations rather than the usual  required to perform the full matrix diagonalization of the Hamiltonian in eq S3.

To account for the influence of structural disorder, we carried out additional centriole simulations, introducing variability into the values of Trp peak excitation energies to test the ensemble’s robustness to static disorder (Figure 5, bottom panel). We considered disorder in the on-site energy of each Trp transition dipole using a random uniform distribution of energies within the range given by *E*_0_ ± *W*/2 for each emitter, introducing *W* as the disorder parameter. The bottom panel of Figure 5 shows the scaling of the normalized superradiance enhancement max[Γ(*W*)]/max[Γ(0)] for Γ(*W*) = {Γ_*j*_(*W*)} as a function of on-site disorder *W*. The normalization factor 1/max[Γ(0)] is introduced to allow the direct comparison of enhancements of centrioles of varying length . As one can see from the bottom panel of Figure 5, as the centriole length increases, a larger value of disorder strength is required to degrade the superradiance enhancement by the same amount. This demonstrates that the Trp network can exhibit cooperative robustness to disorder with increasing network size. The disorder parameter used to obtain the inset in the bottom panel of Figure 5 was *W* = 200 cm^–1^, which corresponds to the Boltzmann energy k_B_*T* at approximately 288 K, revealing that order-of-magnitude max(Γ_*j*_/γ) enhancements are plausible at physiological temperatures.

### Simulations of MT Bundles in Neuronal Axons

*Axons* in neurons can extend vast distances limited only by the scale of the organism,^41^ ranging from millimeters to meters or longer in mammals and varying from hundreds of nanometers to microns in diameter.^42^ Inside an axon, wall-to-wall MT spacings most frequently range from 20 to 30 nm.^29^ We simulated Trp networks in hexagonal bundles of MTs, spaced 50 nm center-to-center (corresponding to a wall-to-wall MT spacing of ∼25 nm), with bundle diameters ranging from ∼100 nm to ∼0.5 μm and containing 7, 19, 37, 61, or 91 MTs (Figure 6). The resulting superradiance data max(Γ_*j*_/γ) data were well-approximated by a set of curves as a function of merely two variables *N*_MT_ and , where *N*_MT_ is the number of bundled MTs and  is the bundle length, with no free parameters. This allowed us to extrapolate our predictions of max(Γ_*j*_/γ) to larger and longer MT bundles (panel 6f), with projected enhancements approaching ∼7000. Like that of the centriole, the spectra of these bundles span a range of about *E*_0_ ± 100 cm^–1^, with a similar energy band structure in the absence of disorder. For numerical simulations, the Hilbert space dimensions were limited in each case by the available computational resources, which is why the maximum axon lengths diminish as one proceeds from panel 6a to panel 6e. Overall, the results we have obtained from simulations of various hierarchical Trp architectures present the prospect of collective and cooperative UV excitonic states in biological media with different characteristic superradiant maxima and subradiant minima (Table S3).

## Discussion

The fluorescence response from multitryptophan proteins, with different lifetime components conventionally associated to different classes of Trp based on the heterogeneity of local environments,^2,3,13^ becomes even more complicated when considering mega-networks of Trp residues formed by their biological architectures. In this work, we have simulated collective photoexcitonic properties of such extremely large Trp networks in protein structures ranging from individual TuDs and MT segments to MT superarchitectures such as the centriole and neuronal bundles (Figure 1).

Even though the coupling between Trp transition dipoles is relatively weak (∼60 cm^–1^) compared to room-temperature energy (∼200 cm^–1^), the presence of long-range couplings between Trp chromophores can greatly enhance the robustness of the network.^28^ Moreover, a counterintuitive consequence of cooperativity is the fact that the robustness of a system to disorder can increase with the system size. This effect, known as cooperative robustness in the literature, has been investigated theoretically in paradigmatic models.^43−45^ In this work, we have shown that mega-networks of Trp in protein architectures can exhibit cooperative robustness, as shown in the bottom panel of Figure 5. The origin of this effect can be qualitatively explained as a very large decay width, strongly coupling the system with the electromagnetic field. Such strong coupling protects the system from disorder, which must become comparable to the coupling in magnitude to suppress superradiance.

On the other hand, our findings also reveal the fundamental challenges of coherent quantum optical information transfer at ambient temperatures in the presence of static and/or dynamical disorder. Significant disorder can effectively quench collective superradiance effects, even though our fluorescence QY measurements of Trp, TuD, and MTs in aqueous buffer solution suggest that even in thermal equilibrium, such effects survive. Certainly, more robust models are needed to account for exciton–phonon couplings in deformations of the protein scaffold,^46^ as well as for optical pumping of mechanical modes in nonequilibrium structural organization and assembly.^47−49^

MTs are crucial to cytoskeletal regulation and form complex bundles in neuronal tissue. Our studies of axonal MT bundles (Figures 1d, 3c, and 6 and Supporting Information) may have implications for both neuroscience and quantum optics research. Confining a superradiant optical mode to one dimension in a waveguide has been proposed to extend emitter interactions to an extremely long range,^50^ raising the tantalizing possibility that axons might serve as such waveguides between giant superradiant emitters in the brain. MT bundles in axons or those associated with the centrosome complex may satisfy a particular combination of criteria necessary to exhibit these ultralong-range couplings, which are currently being exploited for state-of-the-art chiral nanofiber communications systems.^50−52^

## Conclusions

Although the roles of Trp as a metabolic precursor and a fluorescent reporter have been studied in depth, the implications of large Trp architectures for the photophysical control of biosystems remain largely unexplored. Trp chromophores have been identified for their unique role in UV light sensing in the UVR8 plant photoreceptor,^53^ which is believed to be the first UV light perception system discovered to use a network of Trp chromophores as a funnel to enhance its quantum efficiency.^5^ This utilization of a network of intrinsic amino acids for light sensing marks a significant departure from other photoreceptors, which rely on a separate cofactor (such as flavin adenine dinucleotide in cryptochrome) or pigment (such as chlorophyll in photosynthesis or retinal in rhodopsin) to enable light detection and harvesting. Recent observations of UV light-harvesting from Trp networks in MTs^54^ and of the Trp network as a photoreduction mediator in cryptochrome^14^ are consistent with an emerging picture of extended protein scaffolds that harness the symmetries of hierarchical Trp networks to promote biological function.

Past studies elucidated the physical plausibility of superradiant effects in individual MT geometries of varying lengths,^28^ and in this work, we extend these findings to study Trp networks of vastly increased scale, revealing how collective and cooperative quantum effects might manifest in cytoskeletal networks and other protein aggregates associated with diverse cellular structures and organelles. We have also analyzed the collective quantum optical response of MT bundles present in neuronal axons, where photons from brain metabolic activity could be absorbed rapidly via superradiant states for ultrafast information transfer.

Our work highlights essential features of Trp chromophore networks in large aggregates of proteins forming biomolecular superarchitectures such as the centriole (Figures 1c, 3, and 5), axoneme (Figure S3), and MT bundles in neurons (Figures 1d, 3, and 6). Specifically, by analyzing the coupling with the electromagnetic field of mega-networks of Trp present in these biologically relevant architectures, we find the emergence of collective quantum optical effects, namely, superradiant and subradiant eigenmodes. Our analysis has been done using a radiative Hamiltonian (see eq S3 in the Supporting Information) in the single-excitation limit, which is reasonable given the biological milieu of ultraweak photon emissions. The presence of collective superradiant eigenmodes in such a wide variety of biological complexes—and their observed manifestation in increasing QYs for larger hierarchies of proteins—suggests that this collective ultraviolet response would be exploitable in vivo.

Exceptionally bright superradiant states in these biocomplexes may facilitate the absorption and energy transfer of UV photoexcitations in an intensely oxidative environment, where electronically excited molecular species emit light quanta in this wavelength regime. In this manner, superradiant states promoting enhanced QYs for large biological architectures may serve a photoprotective role in pathological conditions such as Alzheimer’s disease and related dementias since an enhanced QY implies that a greater portion of the photonic energy absorbed by certain protein aggregates is re-emitted rather than assimilated by those complexes. Such collective and cooperative mechanisms for photoprotection have not been fully explored, even in the case of the black-brown pigment eumelanin, which consists of a mixture of two indole monomers that aggregate to form oligomers of different lengths and geometries. A recent study of eumelanin^55,56^ demonstrated ultrafast energy transport over large distances despite the significant structural and chemical inhomogeneity of the sample, raising the question of whether mega-networks of indole from Trp and neuromelanin can aid in “internal” UV energy downconversion and funneling in the brain. Similarly, the UV superradiance response in mega-networks of Trp could also augment artificial light-harvesting devices to extend and enhance the spectral band of absorption beyond the visible range.

Our theoretical and numerical predictions thus present numerous possibilities for superradiance- and subradiance-enabled metabolic regulation, communication, and control in and between cells (see Table S3) and with external agents that interact with the cytoskeleton at various stages of cellular growth and replication.^57^ We complement the theory and computation with experimental measurements of fluorescence QY in tubulin and MTs, which point to a significant increase from the former to the latter, in line with the numerical predictions. However, caution must be exerted as these QY measurements need to be complemented by lifetime measurements for the (nonexponential) fluorescence decays. Therefore, our work demonstrates that collective and cooperative UV excitations in Trp mega-networks support robust quantum states in protein aggregates, with observed consequences even under thermal equilibrium conditions.

## Supporting Information Available

Distinctions between Hermitian (closed) and non-Hermitian (open) quantum systems; full steady-state spectroscopy data from Trp networks in protein architectures; presentation of the effective non-Hermitian Hamiltonian for our systems of interest, derived from a Lindblad master equation in the single-excitation limit; physical parameters for Trp, including the peak excitation wavelength, transition dipole strength, and radiative decay rate; clarification between the ^1^L_a_ and ^1^L_b_ transition dipoles of Trp; Lorentzian and Gaussian line shape functions used to model homogeneous broadening of the on-site Trp energies observed in steady-state spectra; reconstruction of steady-state absorption and fluorescence spectra of whole proteins from primary amino acid constituents in aqueous solution; synoptic table of superradiant and subradiant features of Trp networks across multiple biological structures considered in this work; simulations of axoneme superradiance; and visualization of the real and imaginary parts of our non-Hermitian Hamiltonian matrices for MTs of varying lengths, represented in the Trp site basis (PDF)

## Footnotes

a Figure 2 of ref (28) shows a calculated shift in Γ/γ to lower energy by ∼100 cm^–1^ as the number of spirals in an MT increases.
