# Supplementary material for: Ultraviolet Superradiance from Mega-Networks of Tryptophan in Biological Architectures
Source: J Phys Chem B. 2024 Apr 19;128(17):4035–46. doi: 10.1021/acs.jpcb.3c07936 (PMC11075083; doi:10.1021/acs.jpcb.3c07936)
Supplement: Supplementary file 1 — jp3c07936_si_001.pdf [file jp3c07936_si_001.pdf]

Supporting Information: Ultraviolet superradiance from mega-networks of tryptophan in biological architectures

N. S. Babcock,[†] G. Montes-Cabrera,[†,‡] K. E. Oberhofer,[¶] M. Chergui,[¶] G. L. Celardo,[§] and P. Kurian[*,†]

†*Quantum Biology Laboratory, Howard University, Washington DC, 20060 USA*

‡*Institute of Physics, Benemérita Universidad Autónoma de Puebla, Puebla, 72570 Mexico*

¶*Lausanne Centre for Ultrafast Science, École Polytechnique Fédérale de Lausanne, Lausanne, CH-1015 Switzerland*

§*Department of Physics and Astronomy, Università degli Studi di Firenze, Florence, 50019 Italy*

E-mail: pkurian@howard.edu

Non-Hermitian open quantum systems

Table S1: Evolution of Hermitian vs. non-Hermitian systems

Hermitian case	non-Hermitian case
• Schrödinger equation	
$\mathrm{i}\frac{d}{dt}\lvert\psi(t)\rangle = \hat{H}\lvert\psi(t)\rangle \quad (\hat{H}^\dagger = \hat{H})$	$\mathrm{i}\frac{d}{dt}\lvert\psi(t)\rangle = \hat{H}_{\text{eff}}\lvert\psi(t)\rangle \quad (\hat{H}_{\text{eff}}^\dagger \neq \hat{H}_{\text{eff}})$<br>$\hat{H}_{\text{eff}} = \hat{H}_0 - \frac{\mathrm{i}}{2}\hat{\Gamma} \quad (\hat{H}_0^\dagger = \hat{H}_0$ and $\hat{\Gamma}$ is a real and symmetric matrix)
• Evolution operator	
$\lvert\psi(t)\rangle = \hat{U}(t)\lvert\psi(0)\rangle$<br>with the unitary $\hat{U}(t) = \exp(-\mathrm{i}\hat{H}t)$ *i.e.* $\hat{U}\hat{U}^\dagger = \hat{U}^\dagger\hat{U} = \mathbb{1}$	$\lvert\psi(t)\rangle = \hat{\mathcal{U}}(t)\lvert\psi(0)\rangle$<br>with the non-unitary $\hat{\mathcal{U}}(t) = \exp(-\mathrm{i}\hat{H}_{\text{eff}}t)$ *i.e.* $\hat{\mathcal{U}}\hat{\mathcal{U}}^\dagger = \hat{\mathcal{U}}^\dagger\hat{\mathcal{U}} = \mathbb{1}\mathrm{e}^{-\Gamma t}$
• Inner product	
$\langle\psi(t)\lvert\psi(t)\rangle = 1$	$\langle\psi(t)\lvert\psi(t)\rangle = \sum_m C_m^{\mathrm{R}} C_m^{\mathrm{L}} \mathrm{e}^{-\Gamma_m t}$
$\langle\psi(t)\rvert = \langle\psi(0)\rvert\hat{U}^\dagger = \langle\psi(0)\rvert\mathrm{e}^{\mathrm{i}\hat{H}t}$	$\langle\psi(t)\rvert = \langle\psi(0)\rvert\hat{\mathcal{U}}^\dagger(t)$
	with $C_m^{\mathrm{R}} = \langle\psi(0)\lvert\mathcal{E}_m^{\mathrm{R}}\rangle$ and $C_m^{\mathrm{L}} = \langle\mathcal{E}_m^{\mathrm{L}}\lvert\psi(0)\rangle$
• Evolution of an ensemble $\hat{\varrho}$	
$\hat{\varrho}(t) = \hat{U}(t)\hat{\varrho}(0)\hat{U}^\dagger(t)$<br>with $\hat{\varrho}(0) = \sum_\alpha w_\alpha \lvert\psi(0)\rangle\langle\psi(0)\rvert$ and $\sum_\alpha w_\alpha = 1$<br>properties: $\forall t$, (i) $\mathrm{tr}(\hat{\varrho}) = 1$, (ii) $\hat{\varrho}^\dagger = \hat{\varrho}$ and (iii) $\hat{\varrho} > 0$	$\hat{\varrho}(t) = \hat{\mathcal{U}}(t)\hat{\varrho}(0)\hat{\mathcal{U}}^\dagger(t)$<br>with $\hat{\varrho}(0) = \sum_\alpha w_\alpha \lvert\psi(0)\rangle\langle\psi(0)\rvert$ and $\sum_\alpha w_\alpha = 1$<br>properties: for $t \neq 0$, (i) $\mathrm{tr}(\hat{\varrho}) \neq 1$, (ii) $\hat{\varrho}^\dagger = \hat{\varrho}$ and (iii) $\hat{\varrho} > 0$
• Liouville equation	
$\frac{d}{dt}\hat{\varrho}(t) = -\mathrm{i}[\hat{H}, \hat{\varrho}]$	$\frac{d}{dt}\hat{\varrho}(t) = -\mathrm{i}[\hat{H}_0, \hat{\varrho}] - \frac{\mathrm{i}}{2}(\hat{\Gamma}\hat{\varrho} + \hat{\varrho}\hat{\Gamma})$

The dynamics of the chromophore network are governed by the time-dependent Schrödinger equation

$$\frac{d\lvert\psi(t)\rangle}{dt} = -\frac{\mathrm{i}}{\hbar}\hat{H}_{\text{eff}}\,\lvert\psi(t)\rangle\,, \tag{S1}$$

which results in a non-unitary evolution given the non-Hermitian nature of the Hamiltonian $\hat{H}_{\text{eff}}$ from Eq. S3 in the SI. If we denote as $\{\lvert\mathcal{E}_j^{\mathrm{R}}\rangle\}$ the right eigenvectors of $\hat{H}_{\text{eff}}$, $\hat{H}_{\text{eff}}\lvert\mathcal{E}_j^{\mathrm{R}}\rangle = \mathcal{E}_j\lvert\mathcal{E}_j^{\mathrm{R}}\rangle$ with the complex eigenvalue $\mathcal{E}_j = E_j - \mathrm{i}\Gamma_j/2$. As the set of right eigenvectors form an orthonormal basis for the Hilbert space, then the state of the system for $t > 0$ can be written as a linear combination of those states: $\lvert\psi(t)\rangle = \sum_j C_j^{\mathrm{L}}\,\mathrm{e}^{-\mathrm{i}\mathcal{E}_j t/\hbar}\lvert\mathcal{E}_j^{\mathrm{R}}\rangle$, where $C_j^{\mathrm{L}} = \langle\mathcal{E}_j^{\mathrm{L}}\lvert\psi(0)\rangle$, $\lvert\psi(0)\rangle$ is the initial state, and $\langle\mathcal{E}_j^{\mathrm{L}}\rvert$ is the left eigenvector corresponding to $\lvert\mathcal{E}_j^{\mathrm{R}}\rangle$. Since the Hamiltonian is symmetric, the left eigenvectors $\langle\mathcal{E}_j^{\mathrm{L}}\rvert$ are defined as the transpose of the right eigenvectors $\lvert\mathcal{E}_j^{\mathrm{R}}\rangle$.

Since the standard inner product used in Hermitian systems, where $\langle\psi(t)\rvert$ is precisely

defined as the conjugate transpose of the respective $|\psi(t)\rangle$, is not applicable here, it is necessary to introduce an alternative definition, commonly known as the Euclidean inner product (or sometimes the "c-product"[1]). The time evolution of the initial $\langle\psi_0|$ is then given by $\langle\psi(t)| = \sum_j \langle\mathcal{E}_j^{\mathrm{L}}| C_j^{\mathrm{R}}\, e^{\mathrm{i}\mathcal{E}_j^* t}$, with $C_j^{\mathrm{R}} = \langle\psi_0|\mathcal{E}_j^{\mathrm{R}}\rangle$ such that $\langle\psi(t)|\psi(t)\rangle = \sum_j C_j^{\mathrm{R}} C_j^{\mathrm{L}}\, e^{-\Gamma_j t}$. This definition allow us to calculate the time average of any observable we wish to study. In Table S1 of the SI we list some of the main differences between Hermitian and non-Hermitian systems.

Table S2: **Steady-state spectroscopy data from tryptophan networks in protein architectures.** Summary of experimental measurements obtained from steady-state spectroscopy of tryptophan, tubulin dimers, and microtubules in BRB80 aqueous buffer solution (see Fig. 2 for complete spectra). Abbreviations: Absorption maximum (abs max), fluorescence maximum (fluo max), fluorescence quantum yield for Trp contributions at 280 nm (QY-Trp @ 280 nm), fluorescence quantum yield at 295 nm (QY-Trp @ 295 nm), and fluorescence bandwidths at full-width half-maximum for excitation at 280 nm (FWHM fluo @ 280 nm) and 295 nm (FWHM fluo @ 295 nm), respectively. Fluorescence QY is determined from excitation either at 280 nm, where contributions from other amino acids have been subtracted, or at 295 nm, where only tryptophan absorbs and the contributions from other residues can be neglected. Note the statistically significant increases in the QY from tubulin to microtubules, in qualitative agreement with Fig. 3a and consistent with what one would expect in the presence of superradiance. The * indicates an average of upper and lower limit values for microtubules, which have been corrected for the scattering background.

sample	abs max (nm)	fluo max (nm)	FWHM fluo @ 280 nm ($\times 10^3\,\mathrm{cm}^{-1}$)	FWHM fluo @ 295 nm ($\times 10^3\,\mathrm{cm}^{-1}$)	QY-Trp @ 280 nm (%)	QY-Trp @ 295 nm (%)
microtubules (MT)	277±0.5	327±0.5	5.5 ± 0.1	5.4 ± 0.1	17.6* ± 2.1	14.7* ± 1.6
tubulin dimers (TuD)	277±0.5	328±0.5	5.6 ± 0.1	5.6 ± 0.1	10.6 ± 0.6	10.9 ± 1.3
tryptophan (Trp)	278±0.5	355±0.5	4.9 ± 0.1	5.0 ± 0.1	12.4 ± 1.1	11.4 ± 1.1

Superradiance phenomena and model of tryptophan quantum optical networks

It is well known in quantum optics that organized networks of quantum two-level systems can exhibit a phenomenon called *superradiance*, also known as superfluorescence. In 1954, Dicke[2] predicted this behavior, which involves a collection of identical light emitters spontaneously

emitting intense coherent radiation. Since then, superradiance has been observed in various physical systems such as molecular aggregates,[3,4] cold atoms,[5] diamond nanocrystals,[6] semiconductor quantum dot ensembles,[7,8] and more recently in both nanocrystal superlattices[9–11] and hybrid perovskite thin films.[12]

Typically, the probability density that a single excited chromophore emits a photon is exponentially distributed and is characterized by a decay rate γ. If there are N emitters, all in the excited state, superradiance theory predicts cooperative emission with $\sim N^2$ times higher peak intensity than that of a single emitter. If the incident radiation is so weak that only one excitation is present, single-excitation superradiance[13] can result. The hallmark of this cooperative quantum effect, where a single excitation is coherently shared by N emitters, is a decay rate that scales proportionally with $N\gamma$.

The analysis of the coupling of the tryptophan network with the electromagnetic field in the single-excitation limit can be effectively made with a radiative Hamiltonian widely used in quantum optics, which allows treatment of systems whose size is much larger than the absorbed wavelength.[13] Superradiant states can assist photon absorption at specific frequencies and enhance excitation transfer to or along other aggregates due to supertransfer processes.[14,15] Moreover, the presence of superradiant (short-lived, bright) states always comes together with the presence of subradiant (long-lived, dark) states, which can be used to store the absorbed excitation energy.

The tryptophan (Trp) networks we consider are modeled as an ensemble of N two-level systems, each characterized by a transition dipole ($\vec{\mu}_n$). The interaction of a network of two-level systems with the electromagnetic field is described by the effective Hamiltonian[16–18]

$$\hat{H}_{\text{eff}} = \hat{H}_0 + \hat{\Delta} - \frac{\mathrm{i}}{2}\,\hat{\Gamma}\,, \tag{S2}$$

where $\hat{H}_0$ represents the sum of the excitation energies of each Trp chromophore, and $\hat{\Delta}$ and $-\mathrm{i}\,\hat{\Gamma}/2$ represent the coupling matrices between chromophores induced by interaction with

the electromagnetic field. The non-Hermiticity in the term $\mathrm{i}\,\hat{\Gamma}/2$ arises from the fact that the photoexcitation can be lost to the field leading to non-unitary evolution (*i.e.*, spontaneous emission).

The effective Hamiltonian in Eq. S2 has been widely used to model light-matter interactions in the limit of a single excitation, which is reasonable given the biological milieu of ultraweak photon emissions. We consider the primary contribution to the spectra due to the collective interactions between all pairs of Trp choromophores contained in these aggregates,[14] such that $\hat{H}_{\text{eff}}$ may be expanded as

$$\hat{H}_{\text{eff}} = \sum_{n=1}^{N} \Big(\hbar\omega_0 - \mathrm{i}\frac{\gamma}{2}\Big) |n\rangle\langle n| + \sum_{\substack{m,\,n=1;\\ m\neq n}}^{N} \Big(\Omega_{mn} - \mathrm{i}\frac{\Upsilon_{mn}}{2}\Big) |m\rangle\langle n| \,, \tag{S3}$$

where $E_0 = \hbar\omega_0$ is the excitation energy and $\gamma = 4\mu^2 k_0^3/3$ is the spontaneous emission rate of the Trp transition dipole moment $\mu = |\vec{\mu}|$. The angular wavenumber is $k_0 = 2\pi/\lambda$, where λ is the wavelength of the incident light required to bring the Trp chromophore from the ground state to the excited state. Matrix elements Ω_{mn} and Υ_{mn} represent the couplings between the N enumerated Trp transition dipoles in the network, induced by interaction with the electromagnetic field:[14,19]

$$\Omega_{m,n} = \frac{3\gamma}{4}\Bigg\{ -\left[\hat{\mu}_m\cdot\hat{\mu}_n - (\hat{\mu}_m\cdot\hat{r}_{mn})(\hat{\mu}_n\cdot\hat{r}_{mn})\right]\frac{\cos(k_0 r_{mn})}{k_0 r_{mn}} + \left[\hat{\mu}_m\cdot\hat{\mu}_n - 3(\hat{\mu}_m\cdot\hat{r}_{mn})(\hat{\mu}_n\cdot\hat{r}_{mn})\right]\left[\frac{\sin(k_0 r_{mn})}{(k_0 r_{mn})^2} + \frac{\cos(k_0 r_{mn})}{(k_0 r_{mn})^3}\right]\Bigg\},$$

$$\Upsilon_{m,n} = \frac{3\gamma}{2}\Bigg\{ \left[\hat{\mu}_m\cdot\hat{\mu}_n - (\hat{\mu}_m\cdot\hat{r}_{mn})(\hat{\mu}_n\cdot\hat{r}_{mn})\right]\frac{\sin(k_0 r_{mn})}{k_0 r_{mn}} + \left[\hat{\mu}_m\cdot\hat{\mu}_n - 3(\hat{\mu}_m\cdot\hat{r}_{mn})(\hat{\mu}_n\cdot\hat{r}_{mn})\right]\left[\frac{\cos(k_0 r_{mn})}{(k_0 r_{mn})^2} - \frac{\sin(k_0 r_{mn})}{(k_0 r_{mn})^3}\right]\Bigg\}.$$

Here $\hat{\mu}_m = \vec{\mu}_m/\mu_m$ is the unit dipole moment of the mth Trp, and $\hat{r}_{mn} = (\vec{r}_n - \vec{r}_m)/r_{mn}$,

where $r_{mn} = |\vec{r}_n - \vec{r}_m|$ stands for the distance between the mth and n^{th} Trps.

The eigenvalues of the complex symmetric matrix in Eq. S3 can be decomposed into real and imaginary parts, which respectively designate the excitation energies $\{E_j\}$ and decay rates $\{\Gamma_j\}$ of the fluorescent ensemble the matrix describes. Unlike Hermitian operators used to represent systems of bound states, Eq. S3 describes a set of scattering *resonances*.

The values for the physical parameters considered in our analysis are:[14]

- $\lambda = 280\,\text{nm}$ ($E_0 = 35714\ \text{cm}^{-1}$) as the Trp peak excitation wavelength (energy),
- $k_0 = 2\pi E_0 = 2.24 \times 10^{-3}\ \text{Å}^{-1}$ as the angular wavenumber,
- $\mu = 6$ Debye as the strength of the transition dipole between the ground state and the first excited state, with $\mu^2 \approx 181224\ \text{Å}^3\ \text{cm}^{-1}$ (for the conversion, see[14] and for further information on transition dipole states, see below), and
- $\gamma = 4\mu^2 k_0^3/3 = 2.73 \times 10^{-3}\,\text{cm}^{-1}$, where $\gamma/\hbar$ is the radiative decay rate of a single Trp molecule, corresponding to the radiative lifetime $\tau \approx 1.9\,\text{ns}$ (for the conversion, see[14]).

Microtubule structures are comprised mainly of the tubulin dimer (Fig. 1), which gives the structures their characteristic spiral-cylindrical shape. Formed from a pair of subunits denoted α and β, tubulin proteins house a diversity of chromophores, such as tryptophan, tyrosine, and phenylalanine residues. The internal structure of these chromophores, in the form of their aromatic moieties, confer upon them quantum optical properties such as their well-characterized transition dipole moments. Thus, these chromophores can be considered like small two- or three-level systems that absorb light of a certain wavelength and emit it, generally at a different wavelength.

When ordered networks of these chromophores interact with the electromagnetic field, their transition dipoles may synchronize coherently to give rise to superradiance. To characterize this behavior in protein systems as large as the functional axonemes (Fig. S3), centrioles (Fig. 5), and neuronal microtubule bundles (Fig. 6) described in this work, appropriate simplification of the quantum degrees of freedom must be performed. First, our

model only includes tryptophan (Trp) chromophores in each protein structure, because its primary electronic transition dipole moment is considerably larger than that of all other amino acids. Second, assuming that the intensity of the incident light is sufficiently weak, we only consider the limit of the single-excitation manifold. Third, we do not consider any higher electronic transition dipole moments nor the vibronic transitions (0-1, 0-2, etc.) of Trp, for reasons described in the next section. This allows us to describe the interaction between the chromophore network and the electromagnetic field by means of the non-Hermitian effective Hamiltonian in Eq. S3, similar to the tight-binding Hamiltonians typically used in solid-state physics and for photosynthetic light-harvesting complexes.[15]

Clarification between the ^1L_a and ^1L_b transition dipoles

The ^1L_a and ^1L_b transition dipoles of cyclic aromatics take their nomenclature from the geometric orientation of the dipole moments with respect to the aromatic plane, where the dipole vectors representing the transitions are centered in the plane such that the ^1L_a vector overlaps with the *atoms*, whereas the ^1L_b vector overlaps with the *bonds* (Fig. S1). While this geometric definition of ^1L_a and ^1L_b is unambiguous in more benzene-like aromatics such as tyrosine (Tyr) and phenylalanine (Phe), the meaning is obscured in indoles such as tryptophan (Trp) where the orthogonal (perpendicular) dipole moments ^1L_a and ^1L_b are angled so that neither one clearly corresponds to a respective "atom" or "bond" axis.

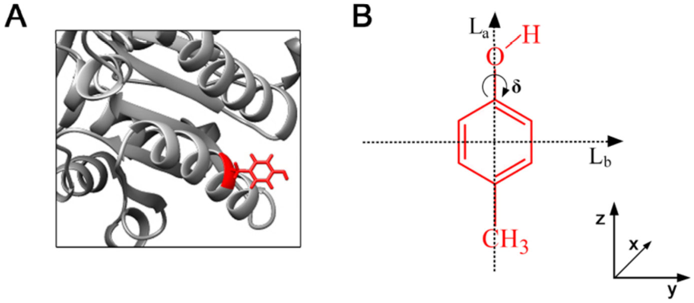

Figure S1: Panel **A** shows protein backbone and tyrosine (Tyr) amino acid, alongside panel **B** showing chemical structure formula of Tyr residue with directions of ^1L_a and ^1L_b transition dipole moments. Reproduced from Ref[20] .

To avoid confusion associated with the aforementioned geometrical ambiguity in Trp, we consider two orthogonal excited states of tryptophan that consist of a larger transition dipole moment that is about twice the strength of the associated smaller one. For the purpose of modeling absorption, we concern ourselves with the 0–0 (purely electronic) transitions rather than the vibronic ones: The 0–0 transition of the larger transition dipole occurs at ~ 300 nm (according to Valeur and Weber[21]), and the 0–0 transition of the smaller one occurs at ~ 290 nm. One should take notice from Valeur and Weber[21] in their Figure 6 that the size of the ^1L_b peak is about half that of the ^1L_a value at that wavelength (~ 290 nm), suggesting that the ^1L_b transition dipole strength is about half that of ^1L_a.[22,23]

Use of the terms ^1L_a and ^1L_b to describe these transitions in Trp persists in the literature, despite the ambiguity of the subscripts in Trp. To clarify this, we may generally consider the ^1L_a electronic transition to be more broadened than the ^1L_b transition in polar solvents because of the larger ^1L_a transition dipole moment, which induces a decrease of the ^1L_a energy below ^1L_b.[23] To include vibronic transitions (0–1, 0–2, *etc.*), the ^1L_a spectrum will have portions that are both lower and higher in energy than that of the ^1L_b spectrum because of this significant line-broadening effect.

On a related note, it is necessary to distinguish the transition spectra of isolated indole from the spectra of the Trp residue itself. For example, Callis [Figure 6 in Ref.[22]] validated calculations for indole ^1L_a and ^1L_b spectra in a polar solvent matrix, by comparing these data to results from Valeur and Weber[21]—but only after re-scaling and translating his calculated values to account for the spectral differences between Trp and indole proper. Thus, Figures 5 and 6 from Valeur and Weber[21] provide actual representations of tryptophan spectra; in particular, the 0–0 transition of ^1L_a (not the peak) is red-shifted with respect to that of ^1L_b.

Absorption and emission spectra

The absorption spectrum indicates the amount of incident electromagnetic radiation absorbed by the chromophores in the network, between a range of energies or frequencies.

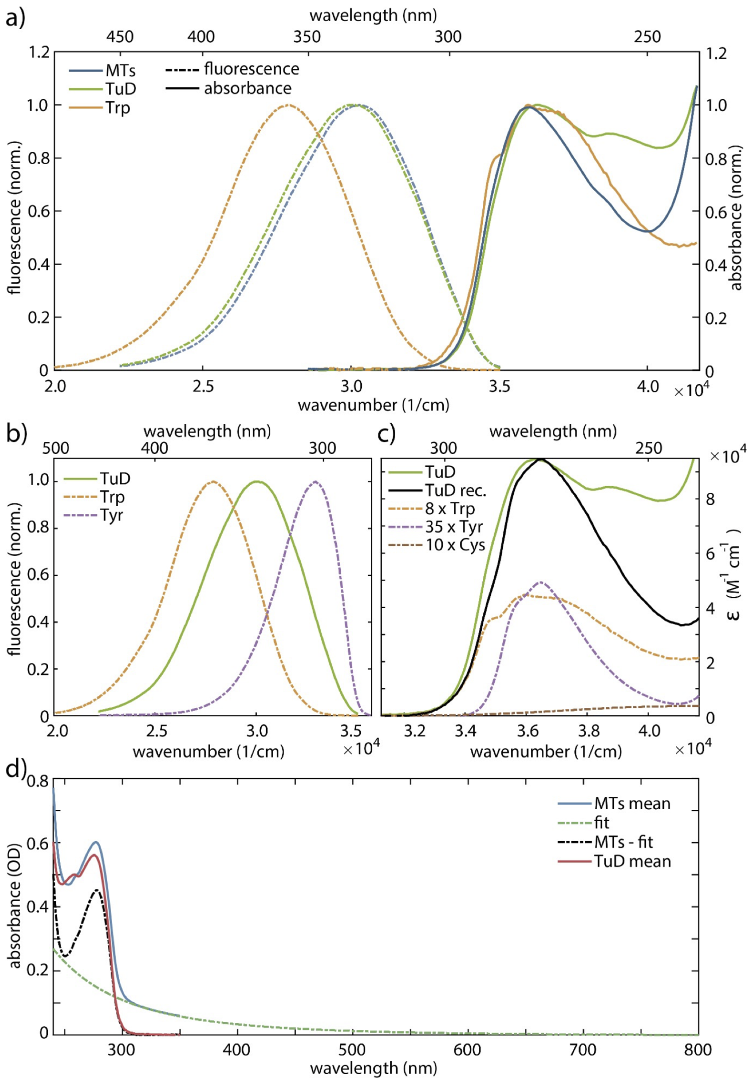

Figure S2: **Steady-state absorption and fluorescence spectra of whole proteins and reconstruction from primary constituents in aqueous solution.** a) Normalized fluorescence spectra (dash-dotted) of MTs (blue), TuD (green) and Trp (orange). Absorption spectra (solid) are normalized to the peak absorption maxima. All spectra are corrected by subtracting background due to solvent absorption scattering. b) Normalized fluorescence spectra of TuD (solid green) and its primary fluorescent constituents Trp (dash-dotted orange) and Tyr (dash-dotted violet). c) Reconstruction of the molar absorption coefficient at 280 nm of TuD (solid black) by adding different contributions of Trp (8 residues, dash-dotted orange), Tyr (35 residues, dash-dotted violet) and Cys (10 residues, dash-dotted brown). The measured absorption spectrum of TuD from a) is added for comparison (solid green). d) Fit of the scattering background of the MTs solution according to a Rayleigh-like model $\propto \lambda^{-4}$ (green dashed). The MTs mean spectrum (blue) was used for determination of the lower limit of the QY, and the background-subtracted spectrum MTs-fit (black dashed) was used for the upper limit of the QY. The mean tubulin spectrum (red) is plotted for comparison.

Following the work of Renger and Marcus,[24] we can define the linear absorption spectrum as the real part of a Fourier-Laplace transform of the dipole-dipole correlation function, which can be expressed as a function of the energy as

$$\mathcal{A}(E) = A \sum_j \Gamma_j D_j(E) \tag{S4}$$

where Γ_j is the decay rate corresponding to the jth eigenstate of the $\hat{H}_{\text{eff}}$, $D_j(E)$ is known as the *lineshape* function, and A is a normalization factor. Under the assumption of Markovian behavior, there is insufficient time for the excitation of vibrational quanta, and a Lorentzian lineshape function centered at E_j is obtained:

$$D_j(E) = \frac{\sigma}{(E - E_j)^2 + \sigma^2} \,. \tag{S5}$$

On the other hand, in the strong coupling regime a Gaussian lineshape is expected:

$$D_j(E) = \exp[-(E - E_j)^2/2\sigma^2]. \tag{S6}$$

The parameter σ measured in units of cm^{-1} is associated with the homogeneous broadening of the on-site chromophore energies. These analytical lineshapes are compared with experimental steady-state absorption and fluorescence spectra for Trp, tubulin dimers, and microtubules in Fig. 2. Additional details on the experimental steady-state spectra are catalogued in Table S2 and in Fig. S2.

Conversely, the fluorescence spectrum indicates the amount of electromagnetic radiation emitted by the chromophores in the network. The expression for the fluorescence emission intensity is obtained by multiplying each lineshape by the corresponding Boltzmann factor

$$I(E) = A' \sum_j \frac{\mathrm{e}^{-E_j/k_B T}}{Z} \Gamma_j D_j(E), \tag{S7}$$

where the partition function $Z = \sum_l \exp[-(E_l)/k_B T]$, and A' is a distinct normalization factor. Here the temperature T is measured in Kelvin (K), and k_B is Boltzmann's constant.

Table S3: Synopsis of superradiant and subradiant features of tryptophan networks (of size N) in biological structures, where $\tau_j = (2\pi c\,\Gamma_j)^{-1}$ is the state lifetime, $\tau_{\text{super}} = (2\pi c\,\max(\Gamma_j))^{-1}$, $\tau_{\text{sub}} = (2\pi c\,\min(\Gamma_j))^{-1}$, and $\mathcal{P}_{\text{super}} = E_{\text{super}}/\tau_{\text{super}}$ is the output power from the maximally superradiant state. MT = microtubule. (Subradiance data is not shown for entries where analytical fits for superradiance have been used.)

Protein Structure, Length in nm	$\frac{\max(\Gamma_j)}{N\gamma}$	τ_{super} (ps)	$\mathcal{P}_{\text{super}}$ (μW)	$\frac{\min(\Gamma_j)}{\gamma}$	τ_{sub} (s)
91-MT Axon, 320 (fit)	0.012	0.428	1.65	—	—
61-MT Axon, 320 (fit)	0.016	0.479	1.47	—	—
Centriole, 400	0.028	0.495	1.42	4.6×10^{-8}	0.042
61-MT Axon, 224	0.020	0.547	1.30	3.6×10^{-10}	5.4
37-MT Axon, 320	0.026	0.602	1.19	2.3×10^{-10}	8.5
91-MT Axon, 152	0.017	0.636	1.13	2.6×10^{-10}	7.5
Axoneme (1JFF), 320	0.031	0.754	0.93	2.8×10^{-10}	6.9
19-MT Axon, 320	0.032	0.769	0.92	9.9×10^{-10}	2.0
7-MT Axon, 640	0.039	0.856	0.81	1.4×10^{-10}	13.9
7-MT Axon, 320	0.071	0.941	0.75	2.8×10^{-9}	0.69
Axoneme (6U42), 320	0.010	2.64	0.26	1.0×10^{-8}	0.19
1 Microtubule, 320	0.120	3.89	0.18	6.21×10^{-8}	0.031

Simulations of axoneme superradiance

The *axoneme* is the microtubule-based structural core of the flagellum or cilium of a eukaryotic cell.[25] It typically contains nine microtubule doublets surrounding a central one (Fig. S3). For comparison, we solved the spectrum of an axoneme modeled as an idealized array of microtubule pairs, again generated from the tubulin protein crystal structure (PDB entry 1JFF).[26] We also considered a more realistic model axoneme based on a ciliary microtubule doublet obtained by cryo-electron microscopy (PDB entry 6U42).[27] Even though the primary function of the axoneme is locomotive and mechanical, both the idealized axoneme and ciliary doublet simulations predicted significant superradiant enhancements in the values of $\max(\Gamma_j/\gamma)$, as shown in the left panel of Fig. S3. The right panel of Fig. S3 shows the energy spectrum of the 1JFF axoneme spread over a range of $E_0 \pm 200\,\text{cm}^{-1}$ around the Trp

peak excitation E_0.

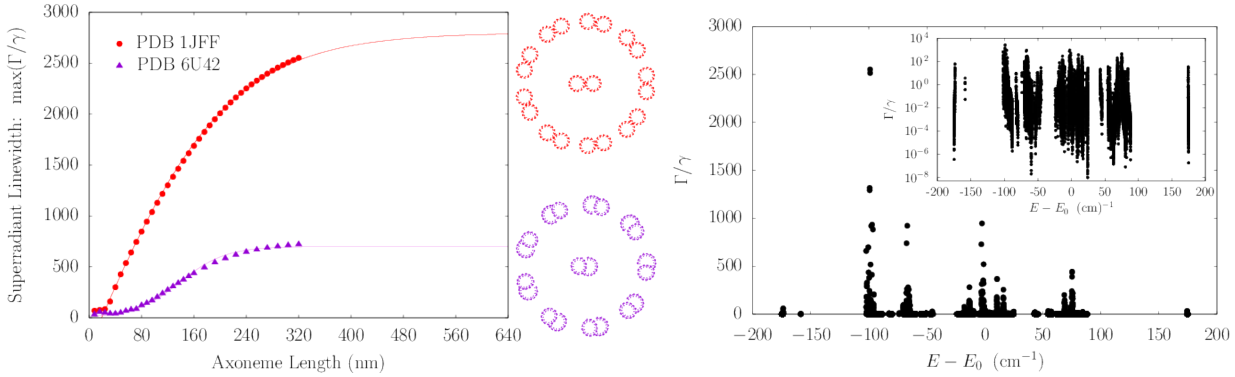

Figure S3: **Ciliary axoneme structures exhibit superradiance, but less so than centrioles and neuronal microtubule bundles of comparable length.** Left panel shows superradiance data points $\max(\Gamma/\gamma)$ calculated from numerical diagonalization of the radiative Hamiltonian in Eq. S3 for a model axoneme (generated using tubulin dimers modeled from PDB entry 1JFF) in red, approximated by the curve $f^{\text{axo}}_{\text{1JFF}}(\ell) = \frac{\lambda n_{\text{D}}}{\ell_0}[(n_{\text{S}} - 2)\tanh(\ell/2n_{\text{S}}\ell_0) - 1]$, where ℓ is the axoneme length along the longitudinal axis (in nm), $\ell_0 = 8\,\text{nm}$ denotes the longitudinal length of a single tubulin spiral, $\lambda = 280\,\text{nm}$ is the excitation wavelength, $n_{\text{D}} = 8$ is the number of Trp dipoles per tubulin dimer, and $n_{\text{S}} = 13$ is the number of dimers per tubulin spiral. Likewise, the $\max(\Gamma/\gamma)$ data points for a more realistic axoneme (constructed using a ciliary doublet from PDB entry 6U42) are shown in violet, fit by the curve $f^{\text{axo}}_{\text{6U42}}(\ell) = \frac{\lambda(n_{\text{S}}-3)}{\ell_0}[\tanh(3\ell/2n_{\text{S}}\ell_0 - 2) + 1]$. Axoneme cross-sections are shown between the two panels as arrays of point dipoles representing the Trp transitions (L_{a}) in the colors red (1JFF) and violet (6U42). Right panel shows the spectrum (Γ/γ *vs* $E - E_0$, where E_0 is the excitation maximum of Trp) of the 320 nm-long model 1JFF axoneme containing 83200 Trp dipoles, plotted on linear and semi-log (inset) scales.

Hamiltonian matrix structure for microtubules

We have plotted the real and imaginary parts of the inter-chromophore tryptophan couplings from the matrix elements H_{ij} of Eq. S3, taking the on-site energies $\Re(H_{jj})$ as zero. Thus, Fig. S4 shows $\Re(H_{ij})$ and $\Im(H_{ij})$ of $\hat{H}$ for a 1-, 10-, and 100-spiral microtubule, respectively.

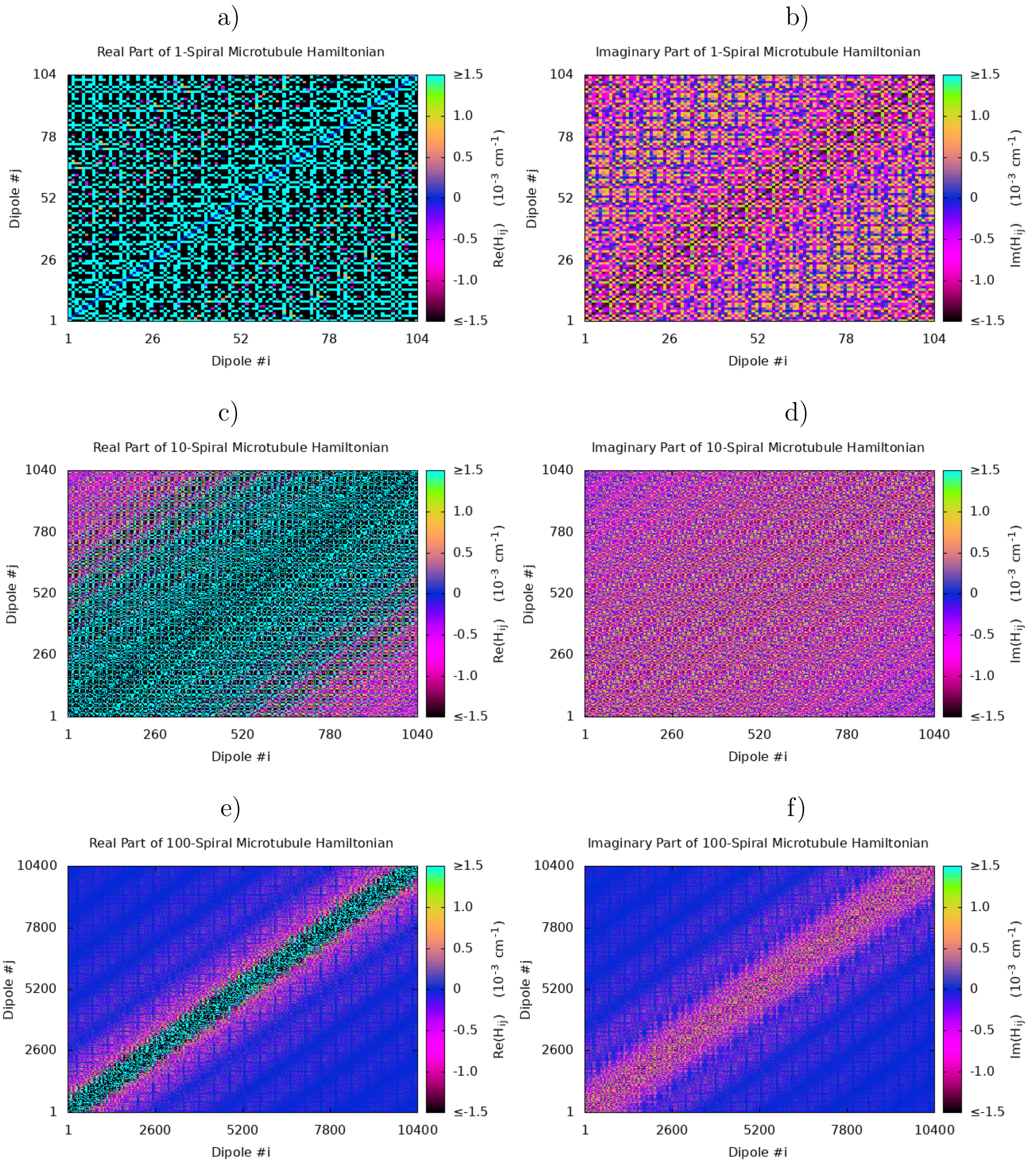

Figure S4: Panels show real and imaginary matrix elements of Hamiltonians for microtubule (MT) tryptophan networks of varying length: a) $\Re(H_{ij})$ for a 1-spiral MT, b) $\Im(H_{ij})$ for a 1-spiral MT, c) $\Re(H_{ij})$ for a 10-spiral MT, d) $\Im(H_{ij})$ for a 10-spiral MT, e) $\Re(H_{ij})$ for a 100-spiral MT, and f) $\Im(H_{ij})$ for a 100-spiral MT.

References

(1) Moiseyev, N. *Non-Hermitian Quantum Mechanics*; Cambridge University Press, 2011.

(2) Dicke, R. H. Coherence in spontaneous radiation processes. *Physical Review* **1954**, *93*, 99.

(3) De Boer, S.; Wiersma, D. A. Dephasing-induced damping of superradiant emission in J-aggregates. *Chemical Physics Letters* **1990**, *165*, 45–53.

(4) Fidder, H.; Knoester, J.; Wiersma, D. A. Superradiant emission and optical dephasing in J-aggregates. *Chemical Physics Letters* **1990**, *171*, 529–536.

(5) Araújo, M. O.; Krešić, I.; Kaiser, R.; Guerin, W. Superradiance in a Large and Dilute Cloud of Cold Atoms in the Linear-Optics Regime. *Physical Review Letters* **2016**, *117*, 073002.

(6) Bradac, C.; Johnsson, M. T.; van Breugel, M.; Baragiola, B. Q.; Martin, R.; Juan, M. L.; Brennen, G. K.; Volz, T. Room-temperature spontaneous superradiance from single diamond nanocrystals. *Nature Communications* **2017**, *8*, 1–6.

(7) Brandes, T. Coherent and collective quantum optical effects in mesoscopic systems. *Physics Reports* **2005**, *408*, 315–474.

(8) Scheibner, M.; Schmidt, T.; Worschech, L.; Forchel, A.; Bacher, G.; Passow, T.; Hommel, D. Superradiance of quantum dots. *Nature Physics* **2007**, *3*, 106–110.

(9) Rainò, G.; Becker, M. A.; Bodnarchuk, M. I.; Mahrt, R. F.; Kovalenko, M. V.; Stöferle, T. Superfluorescence from lead halide perovskite quantum dot superlattices. *Nature* **2018**, *563*, 671–675.

(10) Philbin, J. P.; Kelly, J.; Peng, L.; Coropceanu, I.; Hazarika, A.; Talapin, D. V.; Rabani, E.; Ma, X.; Narang, P. Room temperature single-photon superfluorescence from a

single epitaxial cuboid nano-heterostructure. *https://arxiv.org/abs/2104.06452 [physics.optics]* **2021**, accessed 2021–04–13.

(11) Cherniukh, I.; Rainò, G.; Stöferle, T.; Burian, M.; Travesset, A.; Naumenko, D.; Amenitsch, H.; Erni, R.; Mahrt, R. F.; Bodnarchuk, M. I.; Kovalenko, M. V. Perovskite-type superlattices from lead halide perovskite nanocubes. *Nature* **2021**, *593*, 535–542.

(12) Findik, G.; Biliroglu, M.; Seyitliyev, D.; Mendes, J.; Barrette, A.; Ardekani, H.; Lei, L.; Dong, Q.; So, F.; Gundogdu, K. High-temperature superfluorescence in methyl ammonium lead iodide. *Nature Photonics* **2021**, *15*, 676–680.

(13) Scully, M. O.; Svidzinsky, A. A. The Super of Superradiance. *Science* **2009**, *325*, 1510–1511.

(14) Celardo, G.; Angeli, M.; Craddock, T.; Kurian, P. On the existence of superradiant excitonic states in microtubules. *New Journal of Physics* **2019**, *21*, 023005.

(15) Gullì, M.; Valzelli, A.; Mattiotti, F.; Angeli, M.; Borgonovi, F.; Celardo, G. L. Macroscopic coherence as an emergent property in molecular nanotubes. *New J. Phys.* **2019**, *21*, 013019.

(16) Spano, F. C.; Mukamel, S. Superradiance in molecular aggregates. *The Journal of Chemical Physics* **1989**, *91*, 683–700.

(17) Spano, F. C.; Kuklinski, J. R.; Mukamel, S. Cooperative radiative dynamics in molecular aggregates. *The Journal of Chemical Physics* **1991**, *94*, 7534–7544.

(18) Akkermans, E.; Gero, A.; Kaiser, R. Photon localization and Dicke superradiance in atomic gases. *Physical Review Letters* **2008**, *101*, 103602.

(19) Grad, J.; Hernandez, G.; Mukamel, S. Radiative decay and energy transfer in molecular aggregates: The role of intermolecular dephasing. *Physical Review A* **1988**, *37*, 3835.

(20) Fornander, L. H.; Feng, B.; Beke-Somfai, T.; Nordén, B. UV transition moments of tyrosine. *The Journal of Physical Chemistry B* **2014**, *118*, 9247–9257.

(21) Valeur, B.; Weber, G. Resolution of the fluorescence excitation spectrum of indole into the ^1L_a and ^1L_b excitation bands. *Photochemistry and Photobiology* **1977**, *25*, 441–444.

(22) Callis, P. R. ^1L_a and ^1Lb transitions of tryptophan: Applications of theory and experimental observations to fluorescence of proteins. *Methods in Enzymology* **1997**, *278*, 113–150.

(23) Lombardi, J. R. Solvatochromic shifts reconsidered: Field-induced mixing in the nonlinear region and application to indole. *Journal of Physical Chemistry A* **1999**, *103*, 6335–6338.

(24) Renger, T.; Marcus, R. A. On the relation of protein dynamics and exciton relaxation in pigment–protein complexes: An estimation of the spectral density and a theory for the calculation of optical spectra. *The Journal of Chemical Physics* **2002**, *116*, 9997–10019.

(25) Ishikawa, T. Axoneme Structure from Motile Cilia. *Cold Spring Harbor Perspectives in Biology* **2017**, *9*, a028076.

(26) Löwe, J.; Li, H.; Downing, K.; Nogales, E. Refined structure of $\alpha\beta$-tubulin at 3.5 Å resolution. *Journal of Molecular Biology* **2001**, *313*, 1045–1057.

(27) Ma, M.; Stoyanova, M.; Rademacher, G.; Dutcher, S. K.; Brown, A.; Zhang, R. Structure of the Decorated Ciliary Doublet Microtubule. *Cell* **2019**, *179*, 909–922.
